# Balancing act: Europeans' privacy calculus and security concerns in online CSAM detection

**DOI:** 10.3389/fdata.2025.1477911

**Published:** 2025-01-22

**Authors:** Răzvan Rughiniş, Simona-Nicoleta Vulpe, Dinu Ţurcanu, Daniel Rosner

**Affiliations:** ^1^Faculty of Automatic Control and Computers, National University for Science and Technology Politehnica Bucharest and Academy of Romanian Scientists, Bucharest, Romania; ^2^Faculty of Sociology and Social Work, University of Bucharest and Research Institute of the University of Bucharest (ICUB), Bucharest, Romania; ^3^Faculty of Electronics and Telecommunications and National Institute of Innovations in Cybersecurity “CYBERCOR”, Technical University of Moldova, Chișinău, Moldova; ^4^Faculty of Automatic Control and Computers, National University for Science and Technology Politehnica Bucharest, Bucharest, Romania

**Keywords:** privacy calculus, privacy concerns, age, CSAM detection, Eurobarometer, regression, DESI, AHDI

## Abstract

This study examines privacy calculus in online child sexual abuse material (CSAM) detection across Europe, using Flash Eurobarometer 532 data. Drawing on theories of structuration and risk society, we analyze how individual agency and institutional frameworks interact in shaping privacy attitudes in high-stakes digital scenarios. Multinomial regression reveals age as a significant individual-level predictor, with younger individuals prioritizing privacy more. Country-level analysis shows Central and Eastern European nations have higher privacy concerns, reflecting distinct institutional and cultural contexts. Notably, the Digital Economy and Society Index (DESI) shows a positive association with privacy concerns in regression models when controlling for Augmented Human Development Index (AHDI) components, contrasting its negative bivariate correlation. Life expectancy emerges as the strongest country-level predictor, negatively associated with privacy concerns, suggesting deep institutional mechanisms shape privacy attitudes beyond individual factors. This dual approach reveals that both individual factors and national contexts are shaping privacy calculus in CSAM detection. The study contributes to a better understanding of privacy calculus in high-stakes scenarios, with implications for policy development in online child protection.

## 1 Introduction

The detection of CSAM has become an increasingly serious and urgent problem in the digital age. As internet usage continues to grow globally, so too does the potential for the creation and distribution of CSAM. Law enforcement agencies and technology companies face significant challenges in identifying and removing this content, which can spread rapidly across various platforms and networks. The evolving nature of online spaces, including encrypted messaging systems and peer-to-peer file sharing, further complicates detection efforts. Additionally, the sheer volume of online content makes manual review impractical, necessitating the development of advanced automated detection systems.

While the importance of combating CSAM is clear, the methods used for detection raise substantial privacy concerns. Many detection techniques involve scanning user content, which can be seen as a form of mass surveillance. This approach may infringe on individual privacy rights and potentially expose sensitive personal information unrelated to CSAM. There is also the risk of false positives, where innocuous content is flagged as suspicious, potentially leading to unwarranted investigations or account suspensions. Furthermore, the use of machine learning algorithms in CSAM detection introduces questions about bias and transparency in the decision-making process. Striking a balance between effective CSAM detection and protecting user privacy remains a significant challenge for policymakers and technology developers.

The European Commission's proposed legislation to combat online CSAM has encountered significant obstacles due to privacy concerns. The controversial bill, often referred to as “chat-control,” has been withdrawn from voting, with the Belgian Presidency calling for further negotiations between Member States (Deconinck, 2024). The proposal, which aimed to introduce mandatory scanning of private online communications, including encrypted messages, has faced widespread criticism from privacy organizations, human rights defenders, and technology sector representatives. Critics argue that the legislation would effectively create a mass surveillance system, undermining end-to-end encryption and compromising user privacy. Concerns range from potential breaches of fundamental privacy rights to technical feasibility issues and the risk of creating vulnerabilities exploitable by malicious actors. A study commissioned by the European Parliament heavily criticized the proposal, concluding that current technological solutions cannot detect CSAM without resulting in high error rates affecting all digital communications (Goujard and Manancourt, 2022). The European Data Protection Supervisor (EDPS) and the European Data Protection Board (EDPB) echoed these concerns, warning of potential indiscriminate and extensive scanning of communications content.

In response to these criticisms, there have been attempts to revise the proposal. The European Parliament's Committee on Civil Liberties, Justice, and Home Affairs (LIBE) voted to remove indiscriminate chat control in favor of targeted surveillance of suspicious individuals and groups. However, recent reports suggest efforts to reintroduce indiscriminate message scanning under the guise of moderation of upload (Goujard and Manancourt, 2022). The withdrawal of the vote by the EU Council in June 2024 reflects the significant pushback from critics, including software vendors (Deconinck, 2024).

The Flash Eurobarometer 532 survey, conducted by the European Commission (2023a) in relation to this controversy, provides valuable information about the public opinion regarding the proposed legislation on online CSAM detection. The survey reveals significant variations in perceptions across different demographic factors and countries within the European Union. According to the report, 73% of EU respondents consider CSAM to be “very” or “fairly widespread” in their country. However, this perception varies considerably across member states, ranging from 37% in Latvia to 86% in Greece. Gender and education level also influence these perceptions, with women and those with lower levels of education more likely to view the problem as widespread. The survey also found strong agreement (ranging from 86% to 96% across countries) that children face increasing risks online. Age plays a role in this perception, with older respondents more likely to strongly agree with this statement. The proportion of those agreeing increases from 89% among 18–24-year-olds to 95% among those over 54.

Regarding the balance between child protection and privacy rights, an overwhelming majority (96%) of respondents believe that the ability to detect child abuse is either more important than or equally important to the right to online privacy. However, there are notable variations across countries and age groups. For instance, 72% of respondents in Italy prioritize child abuse detection over privacy, compared to only 35% in Hungary. Age emerges as a significant factor in this privacy calculus. Older respondents (66% of those over 54) are more likely to prioritize child abuse detection over privacy rights, while younger respondents (50% of 18–24-year-olds) tend to view both as equally important. Education level also influences these views, with less educated respondents more likely to prioritize abuse detection over privacy.

Our research aims to further investigate the influence of social factors such as gender, age, education, and national context on the individuals' privacy calculus regarding online CSAM detection. Building on the findings from the Flash Eurobarometer 532 survey (European Commission, 2023a), we conduct a secondary analysis of these data at the individual level. This analysis will control for perceived prevalence of child sexual abuse, perceived online risks for children, and Internet experience to isolate the specific effects of social factors on attitudes toward privacy in the context of child protection.

We will also extend our study to the country level by incorporating additional indicators like the AHDI and the DESI 2022. This approach will help contextualize individual responses within broader societal and technological frameworks. Our research seeks to provide a deeper understanding of how different demographic groups and national contexts shape public opinion on CSAM detection and privacy rights. These evaluations could inform more effective policy approaches and communication strategies in addressing the challenge of online child protection while respecting privacy concerns.

Thus, this study aims to advance our understanding of privacy calculus in high-stakes scenarios by examining how Europeans balance privacy concerns against child protection measures in the context of online CSAM detection. Our research has three primary objectives. First, we seek to identify how individual-level characteristics—including age, gender, education, and digital experience—shape privacy attitudes specifically in this context, extending previous privacy calculus research beyond routine online interactions. Second, we aim to uncover regional patterns and country-level variations in privacy attitudes across Europe, contributing to a deeper understanding of how national contexts influence privacy decisions in high-stakes scenarios. Third, we investigate how broader social indicators, particularly digital development (DESI) and human development (AHDI), relate to national differences in privacy concerns regarding CSAM detection. By examining both individual and country-level factors, our study seeks to provide a comprehensive analysis of how privacy calculus operates when fundamental rights to privacy intersect with critical child protection measures. This understanding is particularly relevant as policymakers and technology developers work to design effective CSAM detection systems that appropriately balance privacy rights with child protection imperatives.

The remainder of this paper is structured as follows. We begin with a literature review examining current knowledge about privacy calculus, focusing on how individual characteristics and national contexts influence privacy decisions in both routine and high-stakes scenarios. The literature review also explores how privacy attitudes may shift in response to perceived social risks, drawing parallels between CSAM detection and other critical contexts. We then describe our materials and methods, detailing our approach to analyzing Flash Eurobarometer 532 data at both individual and country levels, and explaining our use of DESI and AHDI as country-level indicators. The results section presents our findings in two main parts: first, an individual-level analysis examining how socio-demographic factors and internet experience influence privacy calculus; and second, a country-level analysis exploring regional patterns and the relationship between national development indicators and privacy attitudes. In the discussion section, we interpret these findings within the broader context of privacy research, acknowledge the study's limitations, and suggest directions for future research. We conclude by highlighting the implications of our findings for policy development and the design of privacy-preserving CSAM detection systems.

## 2 Literature review

The privacy calculus model, introduced by Culnan and Armstrong (1999), provides a theoretical framework for understanding how individuals make decisions about sharing personal information online. According to this model, people engage in a cognitive cost-benefit analysis when faced with decisions about disclosing personal information. On one side of this calculus, individuals weigh potential privacy risks such as unauthorized data access, surveillance, or misuse of personal information. On the other side, they consider possible benefits, which might include improved services, enhanced security, or broader social gains. This decision-making process becomes increasingly complex in high-stakes scenarios like CSAM detection, where the benefits extend beyond personal advantages to essential social protections. In such contexts, the privacy calculus involves weighing individual privacy rights against collective child protection measures, adding moral and social dimensions to what might otherwise be a more straightforward personal risk-benefit assessment. The privacy calculus model suggests that when perceived benefits outweigh perceived risks, individuals are more likely to accept potential privacy intrusions. However, this calculation can vary significantly based on individual characteristics, contextual factors such as country-level variations in development and digitalization, and the specific nature of the privacy trade-off being considered.

Our analysis draws upon several theoretical frameworks beyond privacy calculus to understand how individuals and societies navigate privacy decisions in high-stakes contexts. Giddens' (1984) theory of structuration helps explain how individual privacy decisions both shape and are shaped by institutional frameworks, while Beck's (1992) concept of risk society illuminates how technological threats transform decision-making processes in modern societies. Beck's notion of “organized irresponsibility” is particularly relevant, describing how modern institutions collectively manage risks while diffusing accountability (Beck, 1998; Galantino, 2022), a dynamic visible in how different societies approach CSAM detection. Additionally, institutional theory (North, 1990) provides insight into how formal and informal constraints shape privacy attitudes across different national contexts. However, emerging technological challenges in CSAM detection call for integration with newer theoretical perspectives. Lyon's (2018) surveillance theory offers a relevant lens for how digital monitoring affects social relationships and trust, while Nissenbaum's (2010) contextual integrity framework helps understand how privacy norms vary across different social contexts. Floridi's (2018) digital ethics framework is particularly relevant for CSAM detection, as it emphasizes the concept of “infraethics”—the design of environments that make ethical behavior more likely. This perspective helps explain why digital maturity might lead to more sophisticated privacy concerns, as societies develop better infrastructures for balancing surveillance and rights. The emphasis that Floridi puts on information ethics as environmental ethics also shows why different social contexts might require different approaches to CSAM detection. Similarly, Zuboff's (2019) theory of surveillance capitalism explains how commercial surveillance mechanisms might be repurposed for child protection. Her concept of “instrumentarian power,” the ability to modify behavior through ubiquitous digital monitoring, raises important questions about how CSAM detection technologies might reshape social relationships and privacy expectations. Zuboff explains how digital natives develop distinctive responses to surveillance and why younger individuals show distinctive privacy concerns. This generational divide in privacy attitudes could represent what Rughiniş and Flaherty (2022) term a “social bifurcation of reality,” where different social groups develop systematically different interpretations of surveillance technologies and their implications.

Recent research suggests that the emergence of generative AI is fundamentally reshaping public discourse and social perceptions around privacy, rights, and technological competence (Bran et al., 2023). This transformation extends beyond individual privacy concerns to affect broader social dynamics, including how people evaluate AI systems' capabilities and legitimacy. The public debate increasingly centers on complex trade-offs between personal rights and collective benefits, with varying vocabularies of competence emerging to justify different positions on AI deployment (Rughiniş et al., 2024). These evolving perceptions are particularly visible in how people present and interpret their digital identities, with AI-mediated self-presentation offering new ways to negotiate social status and identity (Moga and Rughiniş, 2023). The increasing sophistication of AI systems is creating what some researchers identify as a “privacy paradox” (Kokolakis, 2017), where users simultaneously express concerns about privacy while embracing AI-enhanced tools that potentially compromise it, suggesting a complex recalibration of privacy expectations in digitalized societies.

Research has identified several factors that influence this decision-making process. Prior experience with sharing personal information can increase willingness to disclose, as can the perception that information will be used fairly. The specific context and type of information requested also play crucial roles in shaping privacy decisions. Trust in organizations has been shown to positively impact willingness to share information. Cultural dimensions have been found to affect how users evaluate privacy risks and social benefits, particularly in the context of social networking sites. Trepte et al. (2017) observed that individualism/collectivism and uncertainty avoidance significantly influence these evaluations.

Age also appears to be a factor, with Fernandes and Pereira (2021) noting that older users tend to rely more on habits in their privacy decisions, while younger users employ more rational decision-making processes. While some studies have associated demographic factors like gender and education level with privacy concerns, their direct impact on the privacy calculus remains less clear. Research in this area suggests that the privacy calculus model alone may not fully explain behavior. Cognitive biases, social norms, and other theoretical perspectives contribute to a more comprehensive understanding of privacy attitudes and behaviors.

Interestingly, non-conscious factors, especially habits, have been identified as strong influences on willingness to disclose personal data. However, the importance of these factors can vary depending on the context. For sensitive services such as banking or healthcare, rational considerations tend to play a larger role in decision-making.

Research on privacy concerns, a key component of the privacy calculus besides perceived disclosure benefits, reveals significant influences of various social factors. Age consistently emerges as a crucial factor, with several studies finding that older individuals generally express higher levels of privacy concerns compared to younger people (Lee et al., 2019; Steijn et al., 2016; Zhong et al., 2024). However, this relationship is not always linear. Lee et al. (2019) observed that privacy concerns peaked in young to middle adulthood (20s–40s) before decreasing in older age groups. The influence of age on privacy concerns is complicated. Kezer et al. (2016) found that while older adults had higher privacy concerns, younger adults were more likely to engage in privacy protective behaviors on social media. Similarly, Halperin and Dror (2016) reported that younger generations exhibited higher levels of privacy self-efficacy and more proactive privacy protection behaviors, despite higher levels of self-disclosure online. Dhir et al. (2017) found that privacy concerns had a stronger impact on taking selfies for adults compared to adolescents, particularly for males. Elueze and Quan-Haase (2018) expanded on privacy typologies for older adults, identifying five categories ranging from fundamentalists to cynical experts. They found that privacy literacy, extent of digital media use, and perceived self-efficacy in managing privacy were key factors accounting for differences in attitudes among older adults. Moremen et al. (2024) examined generational differences in understanding privacy terminology, finding that Generation Z demonstrated the best understanding overall, while Generation Alpha and older generations (Baby Boomers and Silent Generation) had lower levels of understanding for most terms. Bordonaba-Juste et al. (2020) also found generational differences in factors influencing payment for cloud services, with older generations placing more importance on ubiquity and data loss protection, while younger users valued access to greater online resources. Ray et al. (2021) compared privacy concerns between older adults and working-age adults, finding that while both groups shared similar desires to control personal information, they differed in specific concerns. Older adults were more concerned about global-scale privacy threats and online banking, while working-age adults were more concerned about social media privacy.

Gender has also been identified as a factor influencing privacy concerns, though findings are somewhat inconsistent. Cho et al. (2009) found that women tended to express greater apprehension about online privacy than men. Lee et al. (2019) observed that this gender difference varied with age, with women under 40 showing higher privacy concerns, while men over 50 exhibited greater concerns. Dhir et al. (2017) reported that females had greater privacy concerns than males across all age groups in their study on selfie behavior.

Education level has been associated with privacy concerns in several studies. Lee et al. (2019) found that higher education levels were associated with higher information privacy concerns, with this effect being stronger for younger individuals. Pereira et al. (2017) similarly reported that higher education levels were associated with more concern about privacy and security of both online and health information.

Internet experience and digital literacy appear to play a significant role in shaping privacy concerns, though the relationship is not straightforward. Bellman et al. (2004) found that privacy concerns decreased with greater internet experience, suggesting a potential habituation effect. This is somewhat supported by Engström et al. (2023), who observed weaker preferences for restrictive online self-disclosure in countries with higher internet penetration. However, Prince et al. (2021) found that individuals with higher privacy literacy expressed greater privacy concerns, indicating that increased awareness might heighten rather than alleviate concerns.

Cultural factors and national context also influence privacy concerns. Lancelot Miltgen and Peyrat-Guillard (2014) identified a north-south divide in European attitudes toward privacy, with different emphases on trust and responsibility. Männiste and Masso (2018) highlighted the role of trust in institutions as a key factor influencing privacy concerns in Estonia.

In crisis or high-stake situations like the COVID-19 pandemic and, potentially, online CSAM detection, the privacy calculus can shift dramatically, altering how social factors influence privacy concerns and disclosure behaviors. Lin et al. (2024) propose a three-dimensional framework for understanding privacy perceptions in such contexts, highlighting how the perceived threat moderates the weighing of privacy risks and benefits.

Age continues to play a significant role in shaping privacy perceptions in high-stake situations, but its influence may differ from everyday scenarios. Lin et al. (2024) found that younger respondents (18–29) were less influenced by perceived risks compared to older groups when considering COVID-19 contact tracing apps. This contrasts with findings from non-crisis contexts where older adults generally express higher privacy concerns (Lee et al., 2019; Steijn et al., 2016). The heightened health risks for older adults during the pandemic may have altered their typical privacy calculus, potentially making them more willing to disclose personal information for perceived health benefits, as suggested by Pereira et al. (2017).

Education and digital literacy, which usually correlate with higher privacy concerns (Prince et al., 2021), may have different effects on the privacy calculus in high-stakes situations. More educated individuals might better understand the public health benefits of data sharing, potentially overriding their privacy concerns. However, their awareness of potential data misuses could lead to more cautious behavior, aligning with findings from Kitkowska et al. (2017) that people tend to perceive privacy concerns as comprehensive models.

Cultural factors and trust in institutions play crucial roles in high-stakes scenarios, as highlighted by Männiste and Masso (2018) and Lancelot Miltgen and Peyrat-Guillard (2014). Kim and Kwan (2021) found significant differences in acceptance of COVID-19 mitigation measures between the U.S. and South Korea, with South Koreans showing higher acceptance due to lower privacy concerns and higher perceived social benefits. Trust in government emerges as a key factor in crisis scenarios. Ioannou and Tussyadiah (2021) noted that people who trusted the government to act in citizens' best interests were more accepting of surveillance measures. This suggests that in high-stakes situations like pandemic response or CSAM detection, trust in institutions might become a more significant factor in the privacy calculus than in everyday online interactions. The framing of privacy issues in crisis situations can significantly influence individual decision-making. Ioannou and Tussyadiah (2021) found that framing surveillance as necessary for public health increased acceptance, even among those with privacy concerns. This aligns with Lin et al.'s (2024) observation that crisis psychology can override typical privacy concerns when privacy is framed as a contextualized strategy during crises.

Moreover, the perceived magnitude of both benefits and risks in crisis situations can alter the typical privacy calculus. Kim and Kwan (2021) observed a trade-off between privacy concerns and perceived social benefits in the context of COVID-19 mitigation measures. This trade-off might similarly apply to other high-stakes scenarios like CSAM detection, where the perceived social benefit of child protection could outweigh individual privacy concerns.

Thus, while extensive research has explored the influence of social factors on privacy concerns in everyday online contexts, there remains a significant gap in understanding how these factors shape the privacy calculus in high-stakes scenarios such as online CSAM detection. Previous studies have examined the role of age, gender, education, and internet experience in routine digital interactions (Lee et al., 2019; Steijn et al., 2016) and during crisis situations like the COVID-19 pandemic (Lin et al., 2024; Ioannou and Tussyadiah, 2021). However, the specific context of CSAM detection presents a unique combination of perceived risks and benefits that may alter typical privacy attitudes and behaviors. Moreover, while research has highlighted the importance of cultural and national contexts in shaping privacy perceptions (Kim and Kwan, 2021; Lancelot Miltgen and Peyrat-Guillard, 2014), there is limited understanding of how these factors operate in the face of technologies addressing severe societal issues like CSAM. By investigating how social factors influence the privacy calculus in high-stakes CSAM detection scenarios and exploring the role of national context in shaping related privacy attitudes and behaviors, this research aims to fill the gap in our understanding of privacy dynamics in contexts where child protection and individual privacy rights intersect.

## 3 Materials and methods

In this study we aim to address the following research questions: How do social factors such as age, gender, education, and internet experience influence the privacy calculus in high-stakes scenarios compared to everyday online interactions? What role does the national context play in shaping privacy attitudes and behaviors when confronted with technologies aimed at addressing the severe social issue of CSAM?

Our selection of social factors for analysis is grounded in previous research on privacy attitudes and behaviors. At the individual level, socio-demographic variables have been consistently identified as significant predictors of privacy concerns. Age has been shown to influence both privacy attitudes and protective behaviors, with studies finding generational differences in privacy understanding (Moremen et al., 2024) and risk perception (Lee et al., 2019). Gender has emerged as a relevant factor, with women often expressing greater privacy concerns in online contexts (Cho et al., 2009). Education level has been associated with varying degrees of privacy concern and different approaches to information disclosure (Lee et al., 2019; Pereira et al., 2017). At the country level, our choice of AHDI and DESI as indicators aims to estimate the contextual factors that shape privacy attitudes in digital societies. DESI captures technological development and digital literacy, which previous research has linked to privacy awareness and behavior (Prince et al., 2021; Bellman et al., 2004). AHDI components relate to broader social characteristics that shape privacy attitudes, as demonstrated by studies showing how institutional trust (Männiste and Masso, 2018) and social development (Lancelot Miltgen and Peyrat-Guillard, 2014) influence privacy concerns. These indices thus provide theoretically grounded measures for examining how national context shapes privacy calculus in the specific case of CSAM detection.

Our study employed a secondary analysis of the Flash Eurobarometer 532 data (European Commission, 2023b), focusing on the privacy calculus item that asked respondents: “Which one, if any, of the following statements comes closest to your view? (ONE ANSWER) (1) The ability to detect child abuse is more important than the right to online privacy; (2) The right to online privacy and the ability to detect child abuse are both equally important; (3) The right to online privacy is more important than the ability to detect child abuse.” We applied case weighting in order to ensure representativeness of the EU population aged 18 and over.

The reliance on secondary analysis of Eurobarometer data presents certain limitations. While this dataset offers valuable information through its large-scale, representative sampling across EU member states, it was not specifically designed to comprehensively examine privacy calculus in CSAM detection. Consequently, several potentially relevant variables—such as prior privacy violations, trust in institutions, technical literacy, or detailed measures of risk perception—are not available in the dataset. These unmeasured factors could significantly influence how individuals balance privacy rights against child protection measures. Despite these constraints, the Flash EB 532 data provides a useful opportunity to examine privacy attitudes across Europe in this specific high-stakes context.

We utilized multinomial regression models to examine the factors influencing respondents' choices among these three options. These models aimed to estimate the predictive relevance of various socio-demographic factors, including gender, age, education level, type of residential community (rural, small/mid-sized town, or large town), parental status, and internet experience. Additionally, we considered the influence of national contexts on these attitudes.

To complement the individual-level analysis, we also conducted a country-level analysis using linear regression. This model examined the relationship between the proportion of people in each country who assigned equal or greater importance to privacy concerns compared to CSAM detection (derived from Flash EB 532 responses) and country-level indicators of digital development (DESI 2022) and human development (AHDI). This two-pronged approach, combining individual-level multinomial regression and country-level linear regression, allowed us to investigate both individual and contextual factors shaping privacy attitudes in the high-stakes scenario of online CSAM detection.

The DESI is a comprehensive measure used by the European Union to evaluate and compare the digital progress of its member states. Developed by the European Commission (2024) and published annually since 2014, DESI assesses four key dimensions of digitalization: human capital, connectivity, integration of digital technology, and digital public services. The human capital dimension examines the population's digital skills, from basic internet usage to advanced capabilities. Connectivity focuses on the quality and availability of broadband infrastructure, including both fixed and mobile networks. The integration of digital technology dimension assesses how businesses adopt digital solutions, including their overall digital intensity and engagement in e-commerce. Finally, the digital public services dimension evaluates the progress of e-government initiatives. By analyzing these four aspects, DESI provides a detailed picture of each country's digital landscape, allowing for cross-country comparisons and tracking of progress over time. DESI scores of the EU countries are available in the Supplementary material.

The AHDI is an enhanced measure of societal progress that builds upon the traditional Human Development Index (de la Escosura, 2024). It incorporates four key dimensions: health, education, standard of living, and civil and political freedom. This multifaceted approach recognizes that human development extends beyond economic indicators, encompassing various aspects of well-being and opportunity. The AHDI assesses health through life expectancy at birth, a measure that, as Rughiniş et al. (2022) argue, serves as a powerful indicator of society's broader organizational capacity to sustain and enhance life, reflecting the effectiveness of multiple interconnected social systems. The index also measures education via average years of schooling, and standard of living using GDP per capita, adjusted logarithmically to account for diminishing returns. Uniquely, it includes a measure of civil and political freedom based on the Liberal Democracy Index (Herre, 2024) from the Varieties of Democracy project. To enable meaningful comparisons, each indicator is normalized to a scale of 0–1. The AHDI is then calculated as the geometric mean of these four normalized indices, giving equal weight to each dimension. This method reduces the ability of high performance in one area to compensate for deficiencies in others, providing a more balanced representation of overall human development. By incorporating civil and political freedom, the AHDI offers a more comprehensive view of human development, reflecting the idea that true progress involves expanding people's choices and opportunities across multiple dimensions of life. AHDI scores of the EU countries are available in the Supplementary material.

The multidimensional structure of DESI creates interactions with various aspects of social development. The human capital dimension of DESI, which measures both basic digital skills and advanced technological capabilities, intersects with educational and workforce development aspects of AHDI. This intersection is important for understanding how digital literacy and technological advancement contribute to broader social outcomes. For instance, countries scoring highly on DESI's human capital component demonstrate more sophisticated approaches to digital governance and privacy protection. The digital public services dimension of DESI reflects the institutional capacity of a country to deliver modern governmental services, directly influencing citizens' trust in and engagement with digital systems. Meanwhile, the connectivity and technology integration measurements provide information into the infrastructure supporting digital development, which can either enable or constrain the effectiveness of social policies, including child protection measures. Understanding these interconnections is important for interpreting how digital development indicators interact with broader human development metrics to influence privacy attitudes and social policy effectiveness.

While AHDI and DESI provide standardized measures for cross-national comparison, their use as country-level predictors introduces certain methodological constraints. As composite indices, both AHDI and DESI aggregate multiple dimensions into single scores, potentially masking important country-specific variations that could influence privacy attitudes in the context of CSAM detection. For instance, DESI combines diverse aspects of digitalization—from basic internet usage to advanced digital public services—which may have different relationships with privacy concerns. Similarly, the aggregation of health, education, economic, and political dimensions in AHDI may obscure specific national characteristics, such as historical experiences with surveillance, cultural attitudes toward child protection, or particular institutional arrangements that could distinctively shape privacy calculus. These indices enable systematic cross-national analysis, but their broad nature means they may not fully capture the diverse socio-cultural and institutional factors that influence individual attitudes toward privacy and child protection in different national contexts.

## 4 Individual-level analysis

Our analysis at the individual level involved four multinomial regression models for the privacy calculus item, each incorporating an increasing number of predictor types. Model 1 included only socio-demographic variables (such as age, gender, education, and residential community type). Model 2 added country as a predictor, allowing for the consideration of national contexts. Model 3 further incorporated digital experience variables, while Model 4, the most comprehensive model, also included perceived prevalence of child abuse and perceived online risks for children. Models 1–3 are included in the Supplementary material (along with descriptive statistics for the variables used in the analysis), while Model 4 is presented below.

The Nagelkerke pseudo R-Squared values provide an indication of the models' predictive capacity, with higher values suggesting better model fit. The values show a consistent increase across the models, from 0.040 for Model 1 to 0.122 for Model 4 (see Table 1). The addition of country as a predictor in Model 2 nearly doubled the Nagelkerke value from 0.040 to 0.076, indicating that national context plays a substantial role in explaining variations in privacy calculus attitudes. This significant increase underscores the importance of considering country-level factors in understanding privacy attitudes.

**Table 1:** Predictive power of multinomial regression models.

**Pseudo R- square**	**Model 1**	**Model 2**	**Model 3**	**Model 4**
	Socio-demographics	Socio-demographics and country	Socio-demographics, country, and digital experience	Socio-demographics, country, digital experience, and perceived prevalence and risks
Cox and Snell	0.031	0.059	0.063	0.094
Nagelkerke	0.040	0.076	0.082	0.122
McFadden	0.021	0.041	0.044	0.067

Source: Authors' analysis of Flash EB 532 data.

The inclusion of digital experience variables in Model 3 resulted in a modest improvement, with the Nagelkerke value increasing to 0.082. This suggests that while digital experience adds some explanatory power, its contribution is relatively small compared to socio-demographic and country-level factors.

Model 4, which incorporated perceived prevalence of child abuse and perceived online risks for children, showed the largest improvement in predictive capacity, with the Nagelkerke value reaching 0.122. This notable increase indicates that perceptions of the problem's severity and associated risks play a crucial role in shaping individuals' privacy calculus in the context of CSAM detection.

Our secondary multinomial regression analysis reveals that gender plays a significant role in shaping individuals' privacy calculus regarding online CSAM detection (see Table 2). While there is no substantial difference between men and women in viewing privacy and child abuse detection as equally important, men are notably more likely than women to prioritize online privacy over child abuse detection. Specifically, men have an odds ratio of 1.372 for valuing privacy more than child abuse detection capabilities, a statistically significant effect. This gender disparity could stem from various factors, including potentially heightened sensitivity to child protection issues among women, a stronger emphasis on personal privacy in online contexts by men, or differing perceptions of online risks between genders.

**Table 2:** Multinomial regression model for the privacy calculus item: “Which one, if any, of the following statements comes closest to your view?” Reference value: “1. The ability to detect child abuse is more important than the right to online privacy.”

**Variable type**	**Variable name**	**Value label**	**2. The right to online privacy and the ability to detect child abuse are both equally important**		**3. The right to online privacy is more important than the ability to detect child abuse**	
			**Sig**.	**Exp(B)**	**Sig**.	**Exp(B)**
Socio-demographics	Gender	Male	0.271	1.034	0.007	1.372
		Female				
	Age	15–24 years	0.000	2.578	0.000	3.514
		25–34 years	0.000	2.226	0.000	3.433
		35–44 years	0.000	1.648	0.000	3.575
		45–54 years	0.000	1.473	0.026	1.627
		55–64 years	0.000	1.347	0.075	1.471
		65 and older				
	Graduation age (from formal education)	Still studying	0.304	1.071	0.010	1.667
		Graduated at 17 or younger	0.883	0.993	0.008	1.547
		Graduated at 18–19	0.132	0.942	0.481	0.894
		Graduated at 20–21	0.187	1.063	0.286	0.810
		Graduated at 22+				
	Type of residential community	Rural or village	0.087	1.071	0.137	1.243
		Small or medium town	0.000	1.133	0.058	1.277
		Large town/city				
	Respondent has children (own, step, adopted)	Has children	0.004	0.898	0.002	0.665
		Does not have children				
Country	FR	France	0.092	1.298	0.118	3.571
	BE	Belgium	0.427	1.150	0.323	2.377
	NL	The Netherlands	0.362	0.858	0.222	2.781
	DE	Germany	0.214	0.826	0.852	0.858
	IT	Italy	0.001	0.584	0.649	1.452
	LU	Luxembourg	0.370	0.673	0.830	1.443
	DK	Denmark	0.074	0.699	0.488	1.870
	IE	Ireland	0.883	1.031	0.550	1.791
	GR	Greece	0.580	0.908	0.698	0.679
	ES	Spain	0.127	1.268	0.182	2.979
	PT	Portugal	0.015	1.530	0.822	1.238
	FI	Finland	0.942	1.015	0.182	3.293
	SE	Sweden	0.283	0.824	0.141	3.511
	AT	Austria	0.883	1.027	0.469	1.899
	CY	Cyprus	0.301	0.686	0.782	1.523
	CZ	Czech Republic	0.008	1.587	0.115	3.812
	EE	Estonia	0.468	1.250	0.327	2.987
	HU	Hungary	0.000	3.343	0.156	3.595
	LV	Latvia	0.404	1.270	0.174	4.048
	LT	Lithuania	0.778	1.072	0.388	2.525
	MT	Malta	0.337	1.516	0.652	2.230
	PL	Poland	0.258	1.196	0.235	2.651
	SK	Slovakia	0.014	1.623	0.066	5.031
	SI	Slovenia	0.757	1.082	0.444	2.256
	BG	Bulgaria	0.673	1.082	0.215	3.004
	RO	Romania	0.000	1.827	0.046	5.239
	HR	Croatia				
Internet experience	How often do you use the internet for personal use in the following activities? Browsing websites	1 = never, 6 = every day	0.000	0.918	0.856	0.989
	...Looking at content on social media network websites and apps (e.g., looking at text, images, videos on Facebook, Twitter, Instagram)	1 = never, 6 = every day	0.207	0.986	0.001	0.881
	...Playing games online	1 = never, 6 = every day	0.157	1.011	0.345	1.029
Perceived prevalence and risks	How widespread do you think the problem of online child sexual abuse is in your country? [3 categories]	Online abuse: very or rather rare	0.000	1.276	0.001	1.781
		Online abuse: rather widespread	0.000	1.176	0.493	0.895
		Online abuse: very widespread				
	To what extent do you agree or disagree with the following statements? Children can safely use the internet without being exposed to harmful content [3 categories]	No harmful content: strongly disagree	0.001	0.847	0.046	1.380
		No harmful content: rather disagree	0.003	0.871	0.452	0.895
		No harmful content: rather or strongly agree				
	...Children can safely use the internet without being approached by adults seeking to harm them [3 categories]	No harmful adults: strongly disagree	0.000	0.820	0.000	0.231
		No harmful adults: rather disagree	0.019	1.111	0.000	0.513
		No harmful adults: rather or strongly agree				
	Children are increasingly at risk online [3 categories]	Children increasingly at risk: strongly or rather disagree	0.000	1.953	0.000	6.594
		Children increasingly at risk: rather agree	0.000	1.320	0.001	1.558
		Children increasingly at risk: strongly agree				

Source: Authors' analysis of Flash EB 532 data.

Age emerges as a highly influential factor in this privacy calculus. The model shows a clear and statistically significant trend across age groups, with younger individuals more likely to prioritize privacy or view it as equally important to CSAM detection. Compared to those aged 65 and older, individuals in the 15–24 age group have 2.578 times higher odds of considering privacy and CSAM detection equally important, and 3.514 times higher odds of prioritizing privacy over CSAM detection. This effect gradually diminishes with age but remains significant for both privacy-oriented answers up to the 45–54 age group, which shows 1.473 times higher odds for equal importance and 1.627 times higher odds for prioritizing privacy.

These age-related differences likely reflect varying perceptions of both privacy risks and the benefits of CSAM detection. Younger individuals, while not necessarily more concerned about privacy in general, appear to weigh it more heavily against CSAM detection. This could be due to several factors. Greater familiarity with digital environments may lead younger people to feel more confident in their ability to navigate online risks, potentially reducing their perceived need for protective measures. Younger individuals might also have a more acute awareness of how privacy infringements could affect their digital lives, given their typically higher online engagement. Conversely, older age groups may place a higher priority on child protection measures, possibly due to different life stages, experiences, or generational values. There could also be age-related differences in understanding or awareness of online CSAM issues, influencing how each group perceives the benefits of detection measures.

The model uses graduation age from formal education as a proxy for educational level, revealing some interesting patterns in how education influences the privacy calculus. However, the effects are less pronounced and consistent compared to those observed for age. For those who view privacy and CSAM detection as equally important, the effects are relatively small and not statistically significant across educational levels. More notable differences emerge when considering those who prioritize privacy over CSAM detection. Individuals who are still studying have 1.667 times higher odds of prioritizing privacy compared to those who graduated at age 22 or older, a statistically significant effect (*p* = 0.010). Similarly, those who graduated at 17 or younger have 1.547 times higher odds of prioritizing privacy, also statistically significant (*p* = 0.008).

This pattern suggests that both those with the least formal education (graduated at 17 or younger) and those still in education are more likely to prioritize privacy over CSAM detection compared to those with higher levels of completed formal education. This could reflect different factors at play. Those still studying, likely younger and immersed in digital environments, may have heightened privacy concerns. For those with less formal education, the preference for privacy might stem from different sources, such as less exposure to information about online child protection issues or different risk perceptions.

The model also examines the influence of residential community type. Residents of small or medium towns show a slight tendency to view privacy and CSAM detection as equally important (odds ratio 1.133, *p* = 0.000) or to prioritize privacy (odds ratio 1.277, *p* = 0.058) compared to those in large towns or cities. Rural residents show similar trends, but with non-significant effects. While these findings hint at potential differences in privacy attitudes across community types, the small effect sizes and lack of consistent statistical significance suggest that residential community plays a relatively minor role in shaping individuals' views on this privacy calculus compared to other factors like age or gender.

The model indicates that having children is a relevant factor in shaping individuals' privacy calculus regarding CSAM detection. For those who view privacy and CSAM detection as equally important, respondents with children have 0.898 times the odds of holding this view compared to those without children (*p* = 0.004). This suggests that parents are slightly less likely to view these issues as equally important. The effect is more pronounced for those who prioritize privacy over CSAM detection. Here, respondents with children have 0.665 times the odds of prioritizing privacy compared to non-parents (*p* = 0.002). This indicates that parents are substantially less likely to prioritize privacy over CSAM detection. This could be attributed to parents' heightened awareness of and concern for child safety issues, potentially making them more accepting of measures aimed at protecting children online, even if these measures might impinge on privacy.

The model also includes three indicators of internet experience: frequency of browsing websites, looking at content on social media, and playing games online. These collectively highlight how internet usage patterns influence the privacy calculus regarding CSAM detection. For browsing websites, the effect is minimal and not statistically significant for both response categories. Social media usage shows a more notable effect, particularly for those prioritizing privacy over CSAM detection. The relatively small effect sizes and lack of statistical significance for most of these indicators suggest that the frequency of internet use itself may not be as influential in shaping attitudes toward privacy vs. CSAM detection as other factors in the model. Instead, the nature of the online engagement (particularly social media use) appears more relevant to how individuals balance these concerns.

The model includes several indicators that measure respondents' perceptions of the gravity of online CSAM and related risks to children. First, the perceived prevalence of online CSAM shows a significant impact. As expected, those who view online abuse as “very or rather rare” have higher odds of both considering privacy equally important (odds ratio 1.276, *p* = 0.000) and prioritizing privacy (odds ratio 1.781, *p* = 0.001) compared to those who see it as very widespread.

Regarding perceptions of children's safety online, those who strongly disagree that children can safely use the internet without exposure to harmful content have lower odds of viewing privacy as equally important (odds ratio 0.847, *p* = 0.001) compared to those who agree. Similarly, those who strongly disagree that children can safely use the internet without being approached by harmful adults have significantly lower odds of prioritizing privacy (odds ratio 0.231, *p* = 0.000).

The perception of increasing online risks for children shows a particularly strong effect. Respondents who strongly or rather disagree that children are increasingly at risk online have much higher odds of both viewing privacy as equally important (odds ratio 1.953, *p* = 0.000) and prioritizing privacy (odds ratio 6.594, *p* = 0.000) compared to those who strongly agree. This suggests that not perceiving an increase in online risks for children is strongly associated with a greater emphasis on privacy.

Collectively, these indicators reveal a consistent pattern: respondents who perceive lower risks and higher safety for children online tend to place greater emphasis on privacy in their calculus. Conversely, those who perceive higher risks and lower safety for children online are more inclined to prioritize CSAM detection over privacy concerns.

The multinomial regression results also provide strong evidence that national context is a crucial predictor of individual evaluations in the privacy calculus for online CSAM detection. Using Croatia as the reference country, we observe significant variations across European nations. Eastern European countries such as Romania, Hungary, and Slovakia demonstrate a notable tendency to prioritize privacy or consider it equally important to child abuse detection. For instance, Romanian respondents are much more likely than Croatians to view privacy as more important or equally important, with high odds ratios for both options. Similarly, Hungarian and Slovak respondents show a stronger inclination toward viewing privacy and child abuse detection as equally important or favoring privacy.

Western European countries also exhibit distinct patterns, albeit with some variations. France, for example, shows a significant tendency for its citizens to prioritize privacy or consider it equally important to child abuse detection compared to Croatians. This suggests that French respondents may place a higher value on privacy rights in this context.

Interestingly, some Southern European countries present a different picture. Italy, for instance, demonstrates lower odds ratios, indicating that Italians are less likely than Croatians to prioritize privacy over child abuse detection. This could reflect cultural or societal differences in how privacy and child protection are valued. Some countries, such as Germany, Ireland, and Austria, do not show statistically significant differences from Croatia. This suggests a degree of similarity in privacy attitudes across certain European nations, possibly indicating shared values or similar approaches to balancing privacy and child protection.

The significant variations in privacy calculus attitudes across European nations, as revealed by our individual-level analysis, underscore the need for a better understanding of the factors driving these differences at the country level. To address this, a country-level analysis that associates these variations with established indices such as DESI and AHDI is useful. By examining how these macro-level indicators correlate with national tendencies in privacy attitudes, we can gain valuable information about the structural and developmental factors that shape privacy calculus in the context of online CSAM detection.

## 5 Country-level analysis

For the country-level analysis, we computed the variable “Privacy concerns,” referring to the proportion of respondents in each country who agreed that “The right to online privacy and the ability to detect child abuse are both equally important,” or that “The right to online privacy is more important than the ability to detect child abuse.”

### 5.1 Regional patterns

The data reveals significant variation in privacy attitudes across European countries (see Table 3). On average, 40%−45% of respondents consider privacy equally or more important than detecting CSAM. Hungary stands out as an outlier, with 64% prioritizing privacy equally or above CSAM detection, the highest proportion among all countries. This contrasts sharply with Italy, where only 26% hold this view.

**Table 3:** Country distribution of privacy concerns across the EU.

	**Privacy concerns in relation to CSAM detection: no**	**Privacy concerns in relation to CSAM detection: yes**	
**Country**	**The ability to detect child abuse is more important than the right to online privacy**	**The right to online privacy and the ability to detect child abuse are both equally important**	**The right to online privacy is more important than the ability to detect child abuse**
	**Row 100%**	**Row 100%**	**Row 100%**
Austria	61%	38%	2%
Belgium	58%	40%	2%
Bulgaria	60%	38%	2%
Croatia	61%	38%	1%
Cyprus	69%	29%	2%
Czech Republic	49%	47%	3%
Denmark	65%	33%	2%
Estonia	52%	45%	3%
Finland	58%	39%	3%
France	58%	40%	2%
Germany	67%	32%	1%
Greece	66%	33%	1%
Hungary	36%	63%	1%
Republic of Ireland	60%	39%	1%
Italy	74%	25%	1%
Latvia	50%	46%	4%
Lithuania	57%	40%	3%
Luxembourg	68%	30%	3%
Malta	53%	47%	0%
Netherlands	64%	34%	2%
Poland	56%	42%	2%
Portugal	51%	48%	1%
Romania	49%	48%	3%
Slovakia	49%	48%	4%
Slovenia	58%	39%	2%
Spain	57%	41%	2%
Sweden	66%	31%	3%

Source: Authors' analysis of Flash EB 532 data.

**Figure 1:**
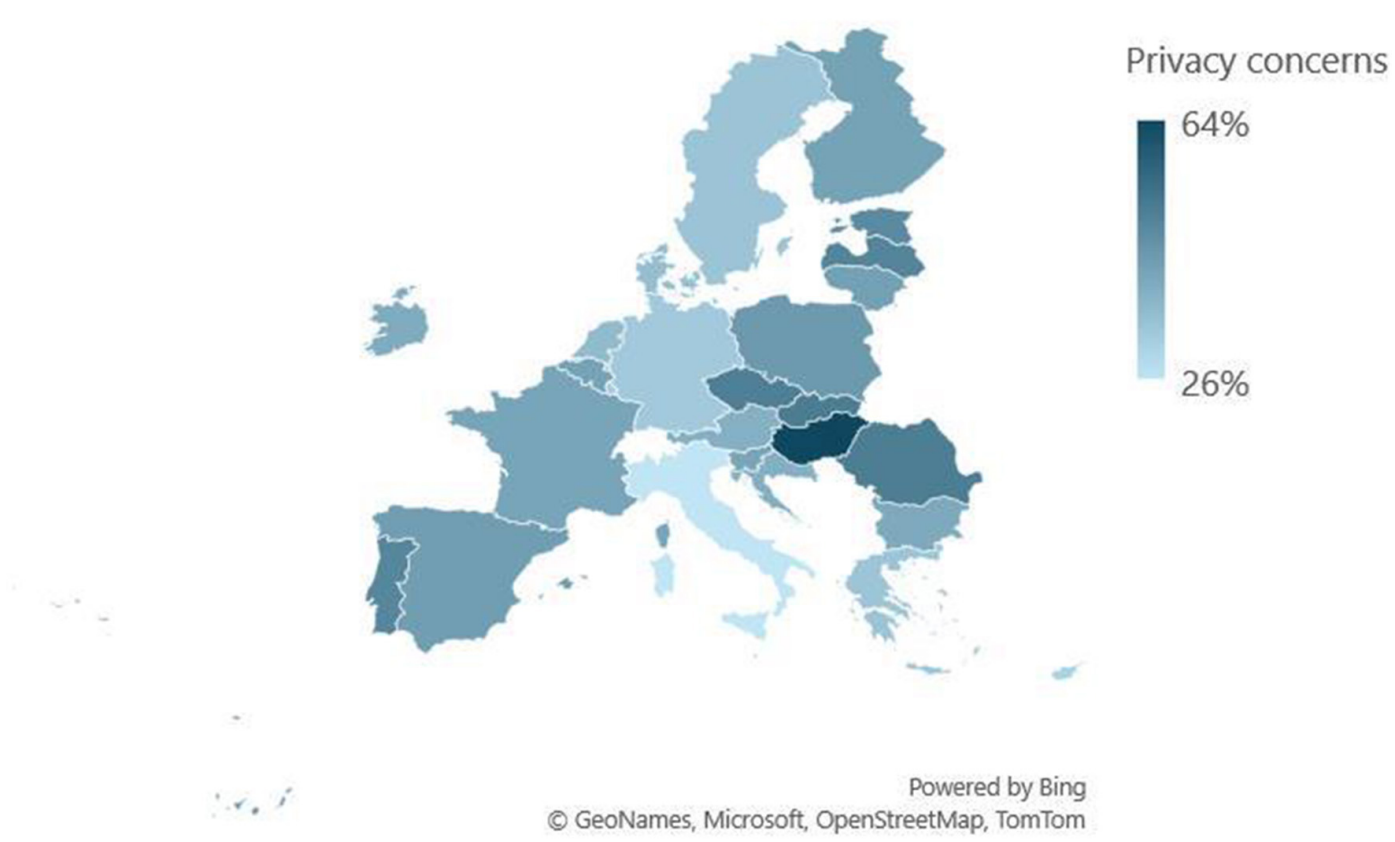
Regional patterns of privacy concerns across the EU. Source: Authors' analysis of Flash EB 532 data.

A regional pattern emerges (see Figure 1) in Central and Eastern Europe, where countries like the Czech Republic, Estonia, Latvia, Romania, and Slovakia show higher privacy concerns, with 45%–51% rating privacy as equally or more important than CSAM detection. On the other side, Western European countries generally demonstrate lower privacy prioritization. Germany, the Netherlands, and Sweden have 33%–36% of respondents valuing privacy equally or above CSAM detection. Southern European countries display mixed results. While Italy shows the lowest privacy concerns, Portugal and Spain have moderate levels, with 49% and 43% respectively prioritizing privacy equally or above CSAM detection. Further research could explore the underlying causes of these variations, including historical experiences with surveillance, digital literacy, and trust in institutions.

### 5.2 Bivariate correlations

**Figure 2:**
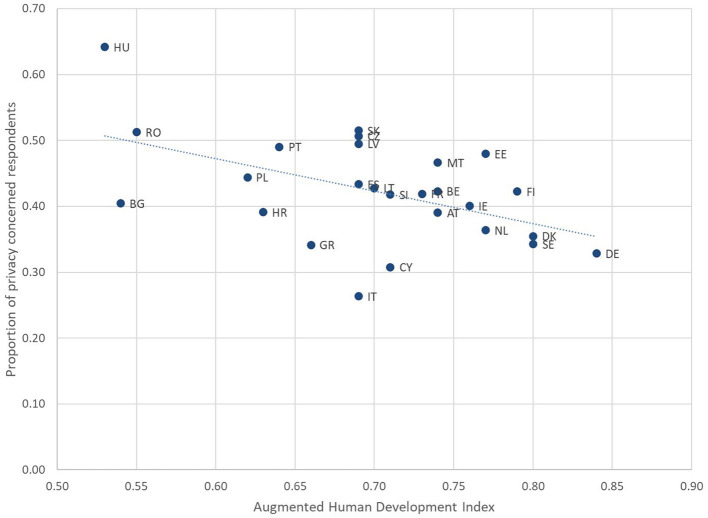
Scatterplot of proportion of privacy concerned respondents vs. AHDI. Source: Authors' analysis of Flash EB 532 and AHDI scores.

The bivariate correlation analysis at country level reveals relationships between privacy concerns and various socioeconomic indicators across countries (see Figure 2). A moderate negative correlation (−0.309) exists between privacy concerns and DESI, suggesting that countries with higher levels of digitalization tend to have lower privacy concerns. Pearson correlation coefficients are available in Table 4.

**Table 4:** Pearson correlation coefficients between privacy concerns and various socioeconomic indicators across countries.

**Variable**		**Privacy concerned**	**DESI**	**Life expectancy**	**Average years of schooling**	**GNI per capita**	**Liberal democracy index**
Privacy concerned	Pearson correlation	1	−0.309	−0.552^**^	−0.011	−0.451^*^	−0.430^*^
	*p*-value		0.116	0.003	0.955	0.018	0.025
DESI	Pearson correlation	−0.309	1	0.663^**^	0.209	0.672^**^	0.681^**^
	*p*-value	0.116		0.000	0.296	0.000	0.000
Life expectancy	Pearson correlation	−0.552^**^	0.663^**^	1	−0.209	0.609^**^	0.492^**^
	*p*-value	0.003	0.000		0.295	0.001	0.009
Average years of schooling	Pearson correlation	−0.011	0.209	−0.209	1	0.224	0.136
	*p*-value	0.955	0.296	0.295		0.261	0.498
GNI per capita	Pearson correlation	−0.451^*^	0.672^**^	0.609^**^	0.224	1	0.551^**^
	*p*-value	0.018	0.000	0.001	0.261		0.003
Liberal democracy index	Pearson correlation	−0.430^*^	0.681^**^	0.492^**^	0.136	0.551^**^	1
	*p*-value	0.025	0.000	0.009	0.498	0.003	

^*^Correlation is significant at the 0.05 level (2-tailed). ^**^Correlation is significant at the 0.01 level (2-tailed). N = 27.

Source: Authors' analysis of Flash EB 532 data.

Life expectancy shows a strong negative correlation (−0.552) with privacy concerns, indicating that countries with higher life expectancy tend to prioritize CSAM detection over privacy. Interestingly, there is a very weak correlation (−0.011) between average years of schooling and privacy concerns, suggesting that education levels in the general population may not significantly influence privacy attitudes in this context. Gross National Income (GNI) per capita demonstrates a moderate negative correlation (−0.451) with privacy concerns. This implies that wealthier countries tend to place less emphasis on privacy relative to CSAM detection. The Liberal Democracy Index also shows a moderate negative correlation (−0.430) with privacy concerns, suggesting that countries with stronger democratic institutions tend to prioritize CSAM detection over privacy.

These correlations indicate that privacy concerns when discussing online CSAM detection are generally lower in more developed, digitalized, and democratic countries. However, the relationships are not uniform across all indicators, and they may be obscured by the relatively high inter-correlation of digitalization and development.

In particular, DESI shows strong positive correlations with several components of AHDI. DESI exhibits a strong positive correlation (0.663) with life expectancy, suggesting that countries with higher levels of digitalization tend to have better health outcomes, and, implicitly, a stronger social organization of the health system. There is a weaker positive correlation (0.209) between DESI and average years of schooling, indicating a limited relationship between digitalization and formal education levels. Still, DESI strongly correlates (0.672) with GNI per capita, implying that more digitalized economies tend to be wealthier. The strongest correlation (0.681) is observed between DESI and the Liberal Democracy Index, suggesting that more digitalized societies tend to have stronger democratic institutions.

These correlations indicate that digital development is closely related with various aspects of human development and governance, particularly economic prosperity and democratic practices. This is why we opted for a linear regression model that can help in disentangling the relative size of associations when controlling for a set of variables.

### 5.3 Linear regression model

The linear regression model at country level estimates the relative importance of various indicators in predicting digital privacy concerns across countries (see Table 5). DESI shows a positive Beta coefficient (0.497), indicating that, when controlling for the AHDI components, higher levels of digitalization are associated with increased privacy concerns. Interestingly, this contrasts with the negative bivariate correlation.

**Table 5:** Linear regression model estimated at country level.

**Variable**	**Beta**	***p*-value**
DESI2022	0.497	0.105
Life expectancy UNDP	−0.651	0.027
Average years of schooling	−0.168	0.413
GNI per capita UNDP	−0.168	0.506
Liberal democracy index	−0.333	0.161

Source: Authors' analysis of Flash EB 532 data.

Life expectancy emerges as the strongest predictor, with a large negative Beta coefficient (−0.651). This is aligned with the earlier correlation findings, suggesting that countries with higher life expectancy tend to prioritize CSAM detection over privacy. Life expectancy in this context likely serves as a proxy for broader patterns of social organization and development rather than directly reflecting health outcomes. It may indirectly measure several aspects of social structure and function. Countries with higher life expectancy often have well-functioning public institutions, including healthcare systems, social services, and regulatory bodies. This institutional capacity may extend to areas of child protection and digital governance. These societies typically exhibit higher levels of social trust, both interpersonal and institutional, which may influence attitudes toward government interventions in digital spaces. Countries with higher life expectancy frequently have more developed welfare states, potentially predisposing populations to accept state interventions for public good. Higher life expectancy can also indicate long-term political and social stability, fostering a greater sense of security and willingness to prioritize child protection over individual privacy.

Societies with higher life expectancy also have larger proportions of older individuals, who may have different attitudes toward digital privacy compared to younger generations. These populations may perceive and prioritize risks differently, potentially viewing online threats to children as more pressing than privacy concerns. These factors, rather than health *per se*, may be driving the observed relationship between life expectancy and attitudes toward privacy vs. CSAM detection.

Average years of schooling and GNI per capita both show modest negative effects (−0.168 for both), indicating that higher education levels and economic development are associated with slightly lower privacy concerns when controlling for the other components of AHDI, albeit to a lesser extent than other factors. Higher education levels may increase awareness of online child exploitation risks, while economic prosperity could shift focus toward protecting vulnerable populations. Both factors might contribute to a sense of security and institutional trust, slightly reducing personal privacy concerns.

The Liberal Democracy Index demonstrates a moderate negative effect (−0.333), suggesting that stronger democratic institutions are associated with lower privacy concerns relative to CSAM detection priorities. Stronger democratic institutions may foster greater trust in government and regulatory bodies, leading citizens to be more accepting of measures aimed at protecting vulnerable groups, such as children. This trust could extend to believing that CSAM detection efforts will be implemented with appropriate safeguards and oversight. Additionally, liberal democracies often have robust public discourse on social issues, potentially raising awareness about online child exploitation and influencing public opinion toward prioritizing child protection.

The Adjusted R Squared (presented in Table 6) shows a moderate predictive power for this linear regression model, explaining 29% of the variation.

**Table 6:** Predictive power of the linear regression model estimated at country level.

**Model**	**R**	**R squared**	**Adjusted R squared**	**Std. error of the estimate**
1	0.653	0.426	0.290	0.068

Source: Authors' analysis of Flash EB 532 data.

## 6 Discussion

The individual-level analysis of privacy calculus in the context of CSAM detection provides several key findings that both align with and extend existing literature on privacy attitudes. Our results show that younger individuals are more likely to prioritize privacy or view it as equally important to CSAM detection compared to older age groups. This contrasts with some previous research (Lee et al., 2019; Steijn et al., 2016) but aligns with the studies of Kezer et al. (2016) and Halperin and Dror (2016). Our study extends these findings to the specific context of CSAM detection.

Gender differences in our analysis reveal that men are more likely to prioritize online privacy over child abuse detection. This contributes to previous research, such as Cho et al. (2009), suggesting that in the context of CSAM detection, gender differences may manifest differently. Furthermore, the influence of education on privacy calculus shows that both those with the least formal education and those still studying are more likely to prioritize privacy compared to those with higher levels of completed formal education. This partially aligns with findings from Lee et al. (2019) and Pereira et al. (2017) but suggests a more complicated relationship in this context.

The analysis of internet experience variables reveals that the nature of online engagement, particularly social media use, is more relevant to privacy calculus than the frequency of internet use alone. This contributes to previous research on the relationship between internet experience and privacy concerns (Bellman et al., 2004; Prince et al., 2021).

The variations observed across European countries underscore the importance of cultural and national contexts in shaping privacy attitudes, extending previous work by Lancelot Miltgen and Peyrat-Guillard (2014) and Kim and Kwan (2021) to this specific scenario. A key finding at country level is the regional pattern observed, with Central and Eastern European countries generally showing higher privacy concerns compared to Western European nations. This aligns with and extends previous research by Lancelot Miltgen and Peyrat-Guillard (2014), who identified regional differences in privacy attitudes across Europe.

The bivariate correlations reveal that privacy concerns are negatively associated with various indicators of national development, including digitalization (DESI), life expectancy, GNI per capita, and the Liberal Democracy Index. This suggests that more developed and democratic countries tend to prioritize CSAM detection over privacy concerns. However, the linear regression model at country level provides a different picture. When controlling for other factors, DESI shows a positive association with privacy concerns, contrasting with the negative bivariate correlation. This highlights the importance of considering multiple factors simultaneously when examining national-level privacy attitudes.

Life expectancy emerges as the strongest predictor of privacy concerns at the country level, with higher life expectancy associated with lower privacy concerns relative to CSAM detection priorities. This finding suggests that life expectancy may serve as a proxy for broader social characteristics that influence privacy attitudes, extending beyond its direct health implications. The negative association between the Liberal Democracy Index and privacy concerns adds to our understanding of how political systems may influence privacy attitudes in high-stakes scenarios. This finding suggests that stronger democratic institutions may foster greater trust in government interventions aimed at child protection.

These country-level findings complement and extend the individual-level analysis. While the individual-level analysis revealed the importance of factors such as age, gender, and education in shaping privacy calculus, the country-level analysis demonstrates how these individual factors operate within broader national contexts. For example, the country-level finding that higher life expectancy (associated with older populations) correlates with lower privacy concerns aligns with the individual-level result that older respondents are less likely to prioritize privacy over CSAM detection.

The somehow divergent effects of digitalization at the individual and country levels (with more frequent social media use associated with higher odds of strong privacy concerns at the individual level, but higher national digitalization associated with lower privacy concerns) indicates that the relevance of digital experience is complicated and warrants further studies.

Our findings highlight the interaction between individual agency and social structure in shaping privacy calculus regarding CSAM detection, illustrating what Giddens (1984) terms the “duality of structure,” where social structures both constrain and enable individual action, while being simultaneously reproduced through that action. This theoretical lens could help explain how personal privacy decisions regarding CSAM detection both shape and are shaped by institutional frameworks. Similarly, Beck's (1992) concept of “risk society” illuminates how modern technological threats, including online risks to children, transform traditional social relations and decision-making processes. The stronger privacy concerns among younger respondents, particularly when controlling for internet experience, suggest that age effects reflect broader generational perspectives on digital rights rather than merely different levels of technological familiarity. This finding aligns with Beck's observation that younger generations in risk societies develop new forms of reflexivity about technological threats.

The observed national variations in privacy attitudes align with North's (1990) institutional theory, suggesting that formal institutions (such as data protection regulations) and informal constraints (such as cultural attitudes toward surveillance) create distinct “rules of the game” that shape privacy calculus across countries. The stronger privacy concerns we found in Central and Eastern European nations particularly reflect what Lyon (2018) terms “surveillance culture,” where historical experiences with monitoring create distinct patterns of resistance or acceptance to new forms of digital surveillance. The age-related variations in privacy attitudes support Nissenbaum's (2010) theory of contextual integrity, suggesting that different generations have developed distinct expectations about appropriate information flows in digital spaces. These theoretical perspectives help explain why privacy calculus in CSAM detection is not simply a matter of individual cost-benefit analysis, but rather a complicated negotiation embedded within broader institutional, cultural, and technological frameworks.

This study makes several contributions to understanding privacy calculus in high-stakes scenarios, with implications for policy development. First, our findings challenge the assumption that privacy concerns necessarily decrease with digital advancement. Instead, we reveal a more complicated relationship where, after controlling for human development factors, higher levels of digitalization actually correspond with increased privacy awareness in CSAM detection contexts. This result suggests that effective policy approaches should evolve with digital maturity rather than assuming simplified trade-offs between privacy and protection. Second, our observation of stronger privacy concerns among younger, digitally engaged populations indicates that future CSAM detection policies must address increasingly sophisticated privacy expectations. Rather than viewing privacy and child protection as competing interests, policymakers should focus on developing technologically advanced solutions that enhance both simultaneously. Third, the significant national variations we identified, particularly regarding institutional trust and historical experiences with surveillance, suggest that CSAM detection policies need careful calibration to local contexts while maintaining consistent European standards. Our findings indicate that successful policy implementation requires an approach at three levels: (1) technical solutions that preserve privacy by design, (2) transparent institutional frameworks that build public trust, and (3) culturally sensitive implementation strategies that account for varying national privacy sensitivities. This multilevel approach to policy development, informed by our empirical findings, could help bridge the current gap between child protection imperatives and privacy concerns in digital spaces.

### 6.1 Limitations

The present study, while providing valuable information about the privacy calculus in the context of CSAM detection, has several limitations that should be considered when interpreting its results. A primary limitation is the use of secondary data from the Flash Eurobarometer 532. While this dataset offers a large, representative sample across European countries, it was not specifically designed for our research questions. As a result, some variables that might be relevant to privacy calculus in this context, such as trust in state institutions or better measures of internet experience, have not been included.

Another methodological limitation of our study is its cross-sectional nature, drawing from a single Eurobarometer survey conducted at one point in time. This temporal constraint means we cannot capture the dynamic nature of privacy attitudes and their evolution in response to rapid technological changes, evolving privacy policies, or shifting social norms regarding online child protection. For instance, the continuous development of AI-based detection technologies, changes in data protection regulations, high-profile privacy breaches, or public debates about encryption could significantly influence how individuals balance privacy rights against child protection measures. The emergence of new online platforms, changing patterns of internet use, and evolving forms of online risks to children may also alter the privacy calculus over time. Longitudinal research would be particularly valuable in understanding how privacy attitudes in this context respond to technological innovations, policy changes, and evolving public discourse about digital privacy and child protection.

While our analysis incorporates broad societal indicators through DESI and AHDI, it does not explicitly account for variations in legal and regulatory frameworks across European countries. Different national approaches to data protection, surveillance laws, child protection regulations, and digital rights enforcement could substantially influence public attitudes toward privacy and CSAM detection. Although all EU countries are required to implement the GDPR and the AI Act, there may be variations regarding online monitoring and national legal frameworks for child protection services. Such regulatory differences could shape both institutional practices and public discourse about privacy rights and child protection, influencing individual privacy calculus. Furthermore, varying levels of regulatory enforcement and different histories of privacy legislation across countries might create distinct contexts that affect how citizens evaluate trade-offs between privacy and child protection measures.

Our study relies on self-reported survey data, which may not fully correspond to individuals' actual behaviors or decision-making in real-world privacy scenarios. Survey responses about privacy attitudes, particularly in the sensitive context of CSAM detection, may be subject to social desirability bias, with respondents potentially overstating their support for child protection measures or their privacy concerns. The well-documented “privacy paradox” (Kokolakis, 2017), where stated privacy preferences often diverge from actual online behaviors, suggests that declared attitudes might not accurately predict how individuals would react to specific CSAM detection measures in practice. Experimental research designs, such as controlled studies of responses to different privacy-preserving detection mechanisms or observational studies of actual user behavior when confronted with child protection measures, would provide valuable complementary evidence to validate and extend our survey-based findings.

Our country-level analysis, while informative, is limited by the relatively small number of countries (27) included in the study. This restricts the statistical power of our analyses. The study's focus on European countries limits its generalizability to other global contexts, where different cultural, legal, and technological landscapes might yield different results.

### 6.2 Future research

Future research could significantly enhance our understanding of privacy calculus in high-stakes scenarios by exploring a wider range of theoretical frameworks, contexts and employing more in-depth methodologies.

A better understanding of this topic would ensue from expanding the theoretical foundation through integration of emerging frameworks in digital ethics, surveillance studies, and child protection. Particularly valuable would be theoretical work examining how different forms of technological risk interact in digital spaces, how privacy norms evolve in response to new protection mechanisms, and how institutional frameworks adapt to balance competing rights in digital contexts. Such theoretical development could help bridge the gap between traditional privacy theories and the specific challenges posed by modern child protection technologies.

A comparative study examining privacy attitudes across different high-stakes scenarios—such as counter-terrorism measures, public health emergencies, and CSAM detection—could reveal how the nature of the perceived threat influences privacy calculus. This could help determine whether the age-related patterns observed in our study are specific to the topic of CSAM or generalizable to other high-stakes contexts. In-depth qualitative interviews or focus groups with individuals from different age groups could provide richer information into the reasoning behind their privacy attitudes, exploring how life experiences, technological familiarity, and generational values shape their perspectives. Longitudinal studies tracking changes in individuals' privacy calculus over time and across different life stages would be particularly valuable in disentangling age effects from cohort effects.

Additionally, experimental studies manipulating the framing of privacy-security trade-offs could help isolate the effects of different factors and mitigate some of the social desirability biases present in survey research. A proposed experimental design would employ a 2 × 2 between-subjects design, manipulating both age group (young adults aged 18–34 vs. older adults aged 50+) and technological framing (privacy-preserving vs. protection-focused). Participants would be randomly assigned to read one of two descriptions of CSAM detection technology: one emphasizing privacy safeguards and encryption protections, the other highlighting child protection benefits and harm prevention. Then, participants would be asked to indicate whether they view CSAM detection capabilities as more important than privacy rights, equally important, or less important than privacy rights, mirroring the Eurobarometer privacy calculus measure. This experimental approach would allow researchers to test causal relationships between technological framing and privacy attitudes while validating current findings about age-related differences in privacy calculus. Conducting such research in Romania, where survey data revealed distinctive privacy concerns, would provide valuable insights into how institutional and cultural contexts influence the effectiveness of different messaging approaches. This experimental extension would complement existing survey findings by testing whether privacy attitudes can be influenced by how CSAM detection technologies are presented, informing more effective communication strategies for balancing privacy and child protection imperatives.

In addition, cross-cultural studies extending beyond Europe could reveal how cultural values and political systems influence privacy calculus in high-stakes scenarios, providing a more global perspective on these issues.

## 7 Conclusion

In conclusion, this study makes several significant contributions to our understanding of privacy calculus in the context of online CSAM detection. By examining both individual-level factors and national contexts, we provide a deeper picture of how Europeans balance privacy concerns against child protection priorities in this high-stakes scenario.

At the individual level, our research extends existing knowledge by demonstrating how age, gender, education, and parental status influence privacy attitudes specifically in the context of CSAM detection. The finding that younger individuals are more likely to prioritize privacy, contrary to some previous research on general privacy concerns, highlights the importance of context-specific studies about individuals' privacy calculus.

Our country-level analysis reveals significant regional variations in privacy attitudes across Europe, contributing to a more geographically aware understanding of privacy concerns. The identification of life expectancy as a strong predictor of national-level privacy attitudes suggests that broader societal characteristics play a crucial role in shaping public opinion on privacy vs. child protection.

The study's dual approach, combining individual and country-level analyses, provides a more holistic view of privacy calculus. Furthermore, our research contributes to the growing body of literature on privacy attitudes in high-stakes scenarios. By focusing on CSAM detection, we shed light on how the gravity of the issue at hand can influence privacy calculus, potentially differing from everyday online privacy concerns.

## Data Availability

Publicly available datasets were analyzed in this study. This data can be found here: Flash Eurobarometer 532 (Protection of Children against Online Sexual Abuse). GESIS Cologne. ZA8763 Data file Version 1.0.0. Available at: https://doi.org/10.4232/1.14214.

## References

[B1] Beck U. (1992). Risk Society: Towards a New Modernity. London: Sage Publications.

[B2] Beck U. (1998). “Politics of risk society,” in The Politics of Risk Society, ed. J. Franklin (Cambridge and Oxford: Polity Press), 9–22.

[B3] Bellman S. Johnson E. J. Kobrin S. J. Lohse G. L. (2004). International differences in information privacy concerns: a global survey of consumers. Inf. Soc. 20, 313–324. 10.1080/01972240490507956

[B4] Bordonaba-Juste M. V. Lucia-Palacios L. Pérez-López R. (2020). Generational differences in valuing usefulness, privacy and security negative experiences for paying for cloud services. Inf. Syst. e-Business Manage. 18, 35–60. 10.1007/s10257-020-00462-8

[B5] Bran E. Rughiniş C. Nadoleanu G. Flaherty M. G. (2023). “The emerging social status of generative AI: vocabularies of AI competence in public discourse,” in Proceedings of the 24th International Conference on Control Systems and Computer Science (CSCS) (Bucharest: IEEE), 391–398. 10.1109/CSCS59211.2023.00068

[B6] Cho H. Rivera-Sánchez M. Lim S. S. (2009). A multinational study on online privacy: global concerns and local responses. New Media Soc. 11, 395–416. 10.1177/1461444808101618

[B7] Culnan M. J. Armstrong P. K. (1999). Information privacy concerns, procedural fairness, and impersonal trust: an empirical investigation. Org. Sci. 10, 104–115. 10.1287/orsc.10.1.10419642375

[B8] de la Escosura L. P. (2024). Augmented Human Development Index [dataset]. Available at: https://ourworldindata.org/grapher/augmented-human-development-index (accessed July 12, 2024).

[B9] Deconinck C. (2024). EU ‘chat-control' plan goes back to drawing board. Brussels Signal. Available at: https://brusselssignal.eu/2024/06/eu-chat-control-plan-goes-back-to-drawing-board/ (accessed August 5, 2024).

[B10] Dhir A. Torsheim T. Pallesen S. Andreassen C. S. (2017). Do online privacy concerns predict selfie behavior among adolescents, young adults and adults? Front. Psychol. 8:815. 10.3389/fpsyg.2017.00815 28588530 PMC5440591

[B11] Elueze I. Quan-Haase A. (2018). Privacy attitudes and concerns in the digital lives of older adults: Westin's privacy attitude typology revisited. Am. Behav. Sci. 62, 1372–1391. 10.1177/0002764218787026

[B12] Engström E. Eriksson K. Björnstjerna M. Strimling P. (2023). Global variations in online privacy concerns across 57 countries. Comput. Hum. Behav. Rep. 9:100268. 10.1016/j.chbr.2023.100268

[B13] European Commission (2023a). Flash Eurobarometer 532 Report: Protection of Children Against Online Sexual Abuse. Available at: https://europa.eu/eurobarometer/surveys/detail/2656 (accessed June 3, 2024).

[B14] European Commission (2023b). Flash Eurobarometer 532 (Protection of Children Against Online Sexual Abuse). GESIS Cologne. ZA8763 Data file version 1.0.0. Available at: 10.4232/1.14214 (accessed June 3, 2024).

[B15] European Commission (2024). The Digital Economy and Society Index (DESI). Available at: https://digital-strategy.ec.europa.eu/en/policies/desi (accessed June 3, 2024).

[B16] Fernandes T. Pereira N. (2021). Revisiting the privacy calculus: why are consumers (really) willing to disclose personal data online? Telemat. Inform. 65:101717. 10.1016/j.tele.2021.101717

[B17] Floridi L. (2018). Soft ethics and the governance of the digital. Philos. Technol. 31, 1–8. 10.1007/s13347-018-0303-9 PMC6191665 30322997

[B18] Galantino M. G. (2022). Organised irresponsibility in the post-truth era: Beck's legacy in today's world at risk. Ital. Sociol. Rev. 12, 971–990. 10.13136/isr.v12i8S.598

[B19] Giddens A. (1984). The Constitution of Society: Outline of the Theory of Structuration. Oxford: Polity Press.

[B20] Goujard C. Manancourt V. (2022). Commission unveils law to fight child sexual abuse online amid swelling privacy fears. Politico. Available at: https://www.politico.eu/article/european-commission-propose-law-fight-child-sexual-abuse-online/ (accessed November 7, 2024).

[B21] Halperin R. Dror Y. (2016). Information privacy and the digital generation gap: an exploratory study. J. Inf. Privacy Secur. 12, 166–180. 10.1080/15536548.2016.1243852

[B22] Herre B. (2024). The ‘varieties of democracy' data: how do researchers measure democracy? Our-WorldInData.org. Available at: https://ourworldindata.org/vdem-electoral-democracy-data (accessed August 5, 2024).

[B23] Ioannou A. Tussyadiah I. (2021). Privacy and surveillance attitudes during health crises: acceptance of surveillance and privacy protection behaviours. Technol. Soc. 67:101774. 10.1016/j.techsoc.2021.101774 34642512 PMC8497958

[B24] Kezer M. Sevi B. Cemalcilar Z. Baruh L. (2016). Age differences in privacy attitudes, literacy and privacy management on Facebook. Cyberpsychol. J. Psychosoc. Res. Cyberspace 10, 1–20. 10.5817/CP2016-1-2

[B25] Kim J. Kwan M. P. (2021). An examination of people's privacy concerns, perceptions of social benefits and acceptance of COVID-19 mitigation measures that harness location information: a comparative study of the US and South Korea. ISPRS Int. J. Geo-Inf. 10:25. 10.3390/ijgi10010025

[B26] Kitkowska A. Meyer J. Wästlund E. Martucci L. (2017). “Is It Harmful? Measuring People's Perceptions of Online Privacy Issues,” in Thirteenth Symposium on Usable Privacy and Security (Santa Clara, CA).

[B27] Kokolakis S. (2017). Privacy attitudes and privacy behaviour: a review of current research on the privacy paradox phenomenon. Comput. Secur. 64, 122–134. 10.1016/j.cose.2015.07.002

[B28] Lancelot Miltgen C. Peyrat-Guillard D. (2014). Cultural and generational influences on privacy concerns: a qualitative study in seven European countries. Euro. J. Inf. Syst. 23, 103–125. 10.1057/ejis.2013.17

[B29] Lee H. Wong S. F. Oh J. Chang Y. (2019). Information privacy concerns and demographic characteristics: data from a Korean media panel survey. Gov. Inf. Q. 36, 294–303. 10.1016/j.giq.2019.01.002

[B30] Lin F. B. Xiong P. Z. Cheng E. W. (2024). A three-dimensional framework of perceiving privacy: a cross-national survey on contact tracing technology and privacy concerns during the COVID-19 pandemic. Comput. Hum. Behav. 152:108047. 10.1016/j.chb.2023.108047

[B31] Lyon D. (2018). The Culture of Surveillance: Watching as a Way of Life. Chichester: Polity Press.

[B32] Männiste M. Masso A. (2018). The role of institutional trust in Estonians' privacy concerns. Stud. Trans. States Soc. 10:22.

[B33] Moga D. A. Rughiniş C. (2023). “Idealized self-presentation through AI avatars: a case study of Lensa AI,” in Proceedings of the 24th International Conference on Control Systems and Computer Science (CSCS) (Bucharest: IEEE), 426–430. 10.1109/CSCS59211.2023.00073

[B34] Moremen C. Hoogsteden J. Birrell E. (2024). Generational differences in understandings of privacy terminology. Proc. Privacy Enhanc. Technol. 3, 70–94. 10.56553/popets-2024-0094

[B35] Nissenbaum H. (2010). Privacy in Context: Technology, Policy, and the Integrity of Social Life. Stanford, CA: Stanford University Press. 10.1515/9780804772891

[B36] North D. C. (1990). Institutions, Institutional Change and Economic Performance. Cambridge: Cambridge University Press. 10.1017/CBO9780511808678

[B37] Pereira S. Robinson J. O. Peoples H. A. Gutierrez A. M. Majumder M. A. McGuire A. L.. (2017). Do privacy and security regulations need a status update? Perspectives from an intergenerational survey. PLoS ONE 12:e0184525. 10.1371/journal.pone.0184525 28926626 PMC5604938

[B38] Prince C. Omrani N. Maalaoui A. Dabic M. Kraus S. (2021). Are we living in surveillance societies and is privacy an illusion? An empirical study on privacy literacy and privacy concerns. IEEE Trans. Eng. Manage. 70, 3553–3570. 10.1109/TEM.2021.3092702

[B39] Ray H. Wolf F. Kuber R. Aviv A. J. (2021). “Warn them” or “just block them”?: investigating privacy concerns among older and working age adults. Proc. Privacy Enhanc. Technol. 2, 27–47. 10.2478/popets-2021-0016

[B40] Rughiniş C. Flaherty M. G. (2022). The social bifurcation of reality: symmetrical construction of knowledge in science-trusting and science-distrusting discourses. Front. Sociol. 7:782851. 10.3389/fsoc.2022.782851 35224088 PMC8864180

[B41] Rughiniş C. Vulpe S. N. Flaherty M. G. Vasile S. (2022). Vaccination, life expectancy, and trust: patterns of COVID-19 and measles vaccination rates around the world. Public Health 210, 114–122. 10.1016/j.puhe.2022.06.027 35963036 PMC9250933

[B42] Rughiniş R. Rughiniş C. Bran E. (2024). “Generative AI and social engines of hate,” in Regulating Hate Speech Created by Generative AI, ed. J. Liebowitz (Boca Raton, FL: Auerbach Publications), 1–18. 10.1201/9781032654829-1

[B43] Steijn W. M. P. Schouten A. P. Vedder A. H. (2016). Why concern regarding privacy differs: the influence of age and (non-) participation on Facebook. Cyberpsychol. J. Psychosoc. Res. Cyberspace 10, 1–12. 10.5817/CP2016-1-3

[B44] Trepte S. Reinecke L. Ellison N. B. Quiring O. Yao M. Z. Ziegele M. (2017). A cross-cultural perspective on the privacy calculus. Soc. Media Soc. 3, 1–13. 10.1177/205630511668803533090108

[B45] Zhong B. Sun T. Zhou Y. Xie L. (2024). Privacy matters: reexamining internet privacy concern among social media users in a cross-cultural setting. Atl. J. Commun. 32, 180–197. 10.1080/15456870.2022.2099548

[B46] Zuboff S. (2019). The Age of Surveillance Capitalism. London: Profile Books.

