# Supplementary material for: Balancing act: Europeans' privacy calculus and security concerns in online CSAM detection
Source: Front Big Data. 2025 Jan 22;8:1477911. doi: 10.3389/fdata.2025.1477911 (PMC11794313; doi:10.3389/fdata.2025.1477911)
Supplement: Supplementary file 1 [file Table_1.docx]

Supplementary Material

# Descriptive statistics for variables included in analysis

**Table 1.** Descriptive values for items included in analysis. Source: Authors’ analysis of Flash EB 532 data.

| **Indicators** | **N** | **N of “Don’t know” responses** | **Min.** | **Max.** | **Mean** | **Std. Deviation** |
| --- | --- | --- | --- | --- | --- | --- |
| Which one, if any, of the following statements comes closest to your view?   1. The ability to detect child abuse is more important than the right to online privacy. 2. The right to online privacy and the ability to detect child abuse are both equally important 3. The right to online privacy is more important than the ability to detect child abuse. | 25613 | 652 | 1 | 3 | 1.41 | 0.53 |
| How often do you use the internet for personal use in the following activities?  Browsing websites (1 = “Never”, 6 = “Every day”) | 26144 | 122 | 1 | 6 | 5.57 | 0.92 |
| How often do you use the internet for personal use in the following activities?  Looking at content on social media network websites and apps (e.g., looking at text, images, videos on Facebook, Twitter, Instagram) (1 = “Never”, 6 = “Every day”) | 26143 | 122 | 1 | 6 | 5.00 | 1.55 |
| How often do you use the internet for personal use in the following activities?  Playing games online (1 = “Never”, 6 = “Every day”) | 26058 | 207 | 1 | 6 | 3.03 | 1.98 |
| How widespread do you think the problem of online child sexual abuse is in your country? (1= “Very rare”, 4= “Very widespread”) | 24575 | 1691 | 1 | 4 | 2.97 | 0.72 |
| Children can safely use the internet without being exposed to harmful content (1 = “Strongly disagree”, 4 = “Strongly agree”) | 25695 | 571 | 1 | 4 | 1.80 | 0.89 |
| Children can safely use the internet without being approached by adults seeking to harm them (1 = “Strongly disagree”, 4 = “Strongly agree”) | 25416 | 850 | 1 | 4 | 1.86 | 0.90 |
| Children are increasingly at risk online (1 = “Strongly disagree”, 4 = “Strongly agree”) | 25918 | 347 | 1 | 4 | 3.44 | 0.67 |
| Gender (1 = “Male”, 2 = “Female”) | 26219 | 46 | 1 | 2 | 1.52 | 0.50 |
| Age | 26265 | 0 | 18 | 95 | 49.20 | 16.47 |
| Graduation age (from formal education) | 25089 | 1176 | 0 | 72 | 20.83 | 4.37 |
| Type of residential community | 26265 | 0 | 1 | 3 | 2.08 | 0.76 |
| Respondent has children (own, step, adopted) | 26265 | 0 | 1 | 2 | 1.73 | 0.45 |

# Multinomial regression models

**Table 2.** Model 1 - Multinomial regression for the privacy calculus item: “Which one, if any, of the following statements comes closest to your view?” Reference value: “1. The ability to detect child abuse is more important than the right to online privacy.” Source: Authors’ analysis of Flash EB 532 data. Source: Authors’ analysis of Flash EB 532 data.

| **Variable type** | **Variable name** | **Value label** | 2. The right to online privacy and the ability to detect child abuse are both equally important | | 3. The right to online privacy is more important than the ability to detect child abuse | |
| --- | --- | --- | --- | --- | --- | --- |
|  |  |  | **Sig.** | **Exp(B)** | **Sig.** | **Exp(B)** |
| Socio-demographics | Gender | Male | 0.000 | 1.124 | 0.000 | 1.875 |
|  |  | Female |  |  |  |  |
|  | Age | 15-24 years | 0.000 | 2.537 | 0.000 | 3.459 |
|  |  | 25-34 years | 0.000 | 2.197 | 0.000 | 4.080 |
|  |  | 35-44 years | 0.000 | 1.632 | 0.000 | 3.478 |
|  |  | 45-54 years | 0.000 | 1.397 | 0.001 | 1.832 |
|  |  | 55-64 years | 0.000 | 1.268 | 0.031 | 1.507 |
|  |  | 65 and older |  |  |  |  |
|  | Graduation age (from formal education) | Still studying | 0.490 | 1.043 | 0.000 | 1.887 |
|  |  | Graduated at 17 or younger | 0.007 | 0.894 | 0.002 | 1.552 |
|  |  | Graduated at 18-19 | 0.000 | 0.870 | 0.164 | 0.820 |
|  |  | Graduated at 20-21 | 0.414 | 1.035 | 0.148 | 0.770 |
|  |  | Graduated at 22+ |  |  |  |  |
|  | Type of residential community | Rural or village | 0.111 | 1.059 | 0.168 | 1.201 |
|  |  | Small or medium town | 0.001 | 1.110 | 0.026 | 1.296 |
|  |  | Large town / city |  |  |  |  |
|  | Respondent has children (own, step, adopted) | Has children | 0.039 | 0.932 | 0.041 | 0.784 |
|  |  | Does not have children |  |  |  |  |

**Table 3.** Model 2 - Multinomial regression for the privacy calculus item: “Which one, if any, of the following statements comes closest to your view?” Reference value: “1. The ability to detect child abuse is more important than the right to online privacy.” Source: Authors’ analysis of Flash EB 532 data. Source: Authors’ analysis of Flash EB 532 data.

| **Variable type** | **Variable name** | **Value label** | 2. The right to online privacy and the ability to detect child abuse are both equally important | | 3. The right to online privacy is more important than the ability to detect child abuse | |
| --- | --- | --- | --- | --- | --- | --- |
|  |  |  | **Sig.** | **Exp(B)** | **Sig.** | **Exp(B)** |
| Socio-demographics | Gender | Male | 0.000 | 1.136 | 0.000 | 1.893 |
|  |  | Female |  |  |  |  |
|  | Age | 15-24 years | 0.000 | 2.633 | 0.000 | 3.528 |
|  |  | 25-34 years | 0.000 | 2.276 | 0.000 | 4.248 |
|  |  | 35-44 years | 0.000 | 1.671 | 0.000 | 3.614 |
|  |  | 45-54 years | 0.000 | 1.422 | 0.001 | 1.882 |
|  |  | 55-64 years | 0.000 | 1.293 | 0.024 | 1.537 |
|  |  | 65 and older |  |  |  |  |
|  | Graduation age (from formal education) | Still studying | 0.409 | 1.053 | 0.000 | 1.953 |
|  |  | Graduated at 17 or younger | 0.773 | 1.013 | 0.000 | 1.984 |
|  |  | Graduated at 18-19 | 0.012 | .912 | 0.307 | 0.862 |
|  |  | Graduated at 20-21 | 0.472 | 1.032 | 0.158 | 0.774 |
|  |  | Graduated at 22+ |  |  |  |  |
|  | Type of residential community | Rural or village | 0.087 | 1.066 | 0.166 | 1.206 |
|  |  | Small or medium town | 0.000 | 1.141 | 0.019 | 1.318 |
|  |  | Large town / city |  |  |  |  |
|  | Respondent has children (own, step, adopted) | Has children | 0.001 | 0.894 | 0.009 | 0.731 |
|  |  | Does not have children |  |  |  |  |
| Country | FR | France | 0.660 | 1.067 | 0.255 | 2.376 |
|  | BE | Belgium | 0.701 | 1.066 | 0.356 | 2.110 |
|  | NL | The Netherlands | 0.136 | 0.789 | 0.395 | 1.950 |
|  | DE | Germany | 0.033 | 0.732 | 0.993 | 0.993 |
|  | IT | Italy | 0.000 | 0.541 | 0.828 | 1.181 |
|  | LU | Luxembourg | 0.231 | 0.612 | 0.704 | 1.717 |
|  | DK | Denmark | 0.174 | 0.773 | 0.262 | 2.557 |
|  | IE | Ireland | 0.893 | 00.974 | 0.671 | 1.475 |
|  | GR | Greece | 0.223 | 0.815 | 0.482 | 0.511 |
|  | ES | Spain | 0.397 | 1.134 | 0.317 | 2.147 |
|  | PT | Portugal | 0.019 | 1.477 | 0.831 | 1.202 |
|  | FI | Finland | 0.979 | 1.005 | 0.155 | 3.271 |
|  | SE | Sweden | 0.056 | 0.721 | 0.173 | 2.950 |
|  | AT | Austria | 0.819 | 0.962 | 0.619 | 1.515 |
|  | CY | Cyprus | 0.158 | 0.611 | 0.913 | 1.168 |
|  | CZ | Czech Republic | 0.012 | 1.519 | 0.092 | 3.796 |
|  | EE | Estonia | 0.272 | 1.367 | 0.194 | 3.890 |
|  | HU | Hungary | 0.000 | 2.936 | 0.192 | 2.972 |
|  | LV | Latvia | 0.184 | 1.409 | 0.065 | 5.564 |
|  | LT | Lithuania | 0.699 | 1.092 | 0.266 | 2.904 |
|  | MT | Malta | 0.503 | 1.312 | 0.799 | 1.557 |
|  | PL | Poland | 0.264 | 1.183 | 0.202 | 2.663 |
|  | SK | Slovakia | 0.021 | 1.540 | 0.057 | 4.746 |
|  | SI | Slovenia | 0.808 | 1.061 | 0.334 | 2.601 |
|  | BG | Bulgaria | 0.775 | 1.052 | 0.166 | 3.139 |
|  | RO | Romania | 0.002 | 1.639 | 0.059 | 4.319 |
|  | HR | Croatia |  |  |  |  |

**Table 4.** Model 3 - Multinomial regression for the privacy calculus item: “Which one, if any, of the following statements comes closest to your view?” Reference value: “1. The ability to detect child abuse is more important than the right to online privacy.” Source: Authors’ analysis of Flash EB 532 data. Source: Authors’ analysis of Flash EB 532 data.

| **Variable type** | **Variable name** | **Value label** | 2. The right to online privacy and the ability to detect child abuse are both equally important | | 3. The right to online privacy is more important than the ability to detect child abuse | |
| --- | --- | --- | --- | --- | --- | --- |
|  |  |  | **Sig.** | **Exp(B)** | **Sig.** | **Exp(B)** |
| Socio-demographics | Gender | Male | 0.000 | 1.157 | 0.000 | 1.814 |
|  |  | Female |  |  |  |  |
|  | Age | 15-24 years | 0.000 | 2.646 | 0.000 | 4.449 |
|  |  | 25-34 years | 0.000 | 2.313 | 0.000 | 5.349 |
|  |  | 35-44 years | 0.000 | 1.730 | 0.000 | 4.486 |
|  |  | 45-54 years | 0.000 | 1.484 | 0.000 | 2.254 |
|  |  | 55-64 years | 0.000 | 1.326 | 0.008 | 1.658 |
|  |  | 65 and older |  |  |  |  |
|  | Graduation age (from formal education) | Still studying | 0.406 | 1.054 | 0.001 | 1.856 |
|  |  | Graduated at 17 or younger | 0.457 | 0.968 | 0.000 | 1.792 |
|  |  | Graduated at 18-19 | 0.008 | 0.905 | 0.148 | 0.809 |
|  |  | Graduated at 20-21 | 0.559 | 1.026 | 0.138 | 0.764 |
|  |  | Graduated at 22+ |  |  |  |  |
|  | Type of residential community | Rural or village | 0.214 | 1.048 | 0.191 | 1.195 |
|  |  | Small or medium town | 0.000 | 1.135 | 0.025 | 1.305 |
|  |  | Large town / city |  |  |  |  |
|  | Respondent has children (own, step, adopted) | Has children | 0.000 | 0.882 | 0.005 | 0.711 |
|  |  | Does not have children |  |  |  |  |
| Country | FR | France | 0.578 | 1.086 | 0.273 | 2.304 |
|  | BE | Belgium | 0.656 | 1.077 | 0.377 | 2.046 |
|  | NL | The Netherlands | 0.167 | 0.801 | 0.407 | 1.920 |
|  | DE | Germany | 0.040 | 0.739 | 0.921 | 0.927 |
|  | IT | Italy | 0.000 | 0.554 | 0.777 | 1.242 |
|  | LU | Luxembourg | 0.282 | 0.643 | 0.664 | 1.859 |
|  | DK | Denmark | 0.156 | 0.763 | 0.285 | 2.449 |
|  | IE | Ireland | 0.959 | 1.010 | 0.640 | 1.536 |
|  | GR | Greece | 0.297 | 0.839 | 0.508 | 0.532 |
|  | ES | Spain | 0.343 | 1.152 | 0.305 | 2.193 |
|  | PT | Portugal | 0.012 | 1.523 | 0.722 | 1.361 |
|  | FI | Finland | 0.847 | 1.038 | 0.136 | 3.467 |
|  | SE | Sweden | 0.107 | 0.757 | 0.154 | 3.111 |
|  | AT | Austria | 0.810 | 0.960 | 0.660 | 1.445 |
|  | CY | Cyprus | 0.211 | 0.646 | 0.853 | 1.304 |
|  | CZ | Czech Republic | 0.008 | 1.556 | 0.087 | 3.879 |
|  | EE | Estonia | 0.239 | 1.402 | 0.176 | 4.126 |
|  | HU | Hungary | 0.000 | 3.022 | 0.162 | 3.216 |
|  | LV | Latvia | 0.142 | 1.466 | 0.054 | 6.054 |
|  | LT | Lithuania | 0.562 | 1.143 | 0.234 | 3.168 |
|  | MT | Malta | 0.444 | 1.367 | 0.732 | 1.816 |
|  | PL | Poland | 0.150 | 1.243 | 0.172 | 2.855 |
|  | SK | Slovakia | 0.014 | 1.587 | 0.052 | 4.923 |
|  | SI | Slovenia | 0.745 | 1.083 | 0.324 | 2.659 |
|  | BG | Bulgaria | 0.650 | 1.085 | 0.146 | 3.334 |
|  | RO | Romania | 0.002 | 1.648 | 0.052 | 4.534 |
|  | HR | Croatia |  |  |  |  |
| Internet experience | How often do you use the internet for personal use in the following activities? Browsing websites | 1 = Never, 6 = Every day | 0.000 | 0.885 | 0.001 | 0.847 |
|  | ...Looking at content on social media network websites and apps (e.g., looking at text, images, videos on Facebook, Twitter, Instagram) | 1 = Never, 6 = Every day | 0.159 | 0.986 | 0.000 | 0.811 |
|  | ...Playing games online | 1 = Never, 6 = Every day | 0.013 | 1.018 | 0.040 | 1.057 |

**Table 5.** Model 4 - Multinomial regression for the privacy calculus item: “Which one, if any, of the following statements comes closest to your view?” Reference value: “1. The ability to detect child abuse is more important than the right to online privacy.” Source: Authors’ analysis of Flash EB 532 data. Source: Authors’ analysis of Flash EB 532 data.

| **Variable type** | **Variable name** | **Value label** | 2. The right to online privacy and the ability to detect child abuse are both equally important | | 3. The right to online privacy is more important than the ability to detect child abuse | |
| --- | --- | --- | --- | --- | --- | --- |
|  |  |  | **Sig.** | **Exp(B)** | **Sig.** | **Exp(B)** |
| Socio-demographics | Gender | Male | 0.271 | 1.034 | 0.007 | 1.372 |
|  |  | Female |  |  |  |  |
|  | Age | 15-24 years | 0.000 | 2.578 | 0.000 | 3.514 |
|  |  | 25-34 years | 0.000 | 2.226 | 0.000 | 3.433 |
|  |  | 35-44 years | 0.000 | 1.648 | 0.000 | 3.575 |
|  |  | 45-54 years | 0.000 | 1.473 | 0.026 | 1.627 |
|  |  | 55-64 years | 0.000 | 1.347 | 0.075 | 1.471 |
|  |  | 65 and older |  |  |  |  |
|  | Graduation age (from formal education) | Still studying | 0.304 | 1.071 | 0.010 | 1.667 |
|  |  | Graduated at 17 or younger | 0.883 | 0.993 | 0.008 | 1.547 |
|  |  | Graduated at 18-19 | 0.132 | 0.942 | 0.481 | 0.894 |
|  |  | Graduated at 20-21 | 0.187 | 1.063 | 0.286 | 0.810 |
|  |  | Graduated at 22+ |  |  |  |  |
|  | Type of residential community | Rural or village | 0.087 | 1.071 | 0.137 | 1.243 |
|  |  | Small or medium town | 0.000 | 1.133 | 0.058 | 1.277 |
|  |  | Large town / city |  |  |  |  |
|  | Respondent has children (own, step, adopted) | Has children | 0.004 | 0.898 | 0.002 | 0.665 |
|  |  | Does not have children |  |  |  |  |
| Country | FR | France | 0.092 | 1.298 | 0.118 | 3.571 |
|  | BE | Belgium | 0.427 | 1.150 | 0.323 | 2.377 |
|  | NL | The Netherlands | 0.362 | 0.858 | 0.222 | 2.781 |
|  | DE | Germany | 0.214 | 0.826 | 0.852 | 0.858 |
|  | IT | Italy | 0.001 | 0.584 | 0.649 | 1.452 |
|  | LU | Luxembourg | 0.370 | 0.673 | 0.830 | 1.443 |
|  | DK | Denmark | 0.074 | 0.699 | 0.488 | 1.870 |
|  | IE | Ireland | 0.883 | 1.031 | 0.550 | 1.791 |
|  | GR | Greece | 0.580 | 0.908 | 0.698 | 0.679 |
|  | ES | Spain | 0.127 | 1.268 | 0.182 | 2.979 |
|  | PT | Portugal | 0.015 | 1.530 | 0.822 | 1.238 |
|  | FI | Finland | 0.942 | 1.015 | 0.182 | 3.293 |
|  | SE | Sweden | 0.283 | 0.824 | 0.141 | 3.511 |
|  | AT | Austria | 0.883 | 1.027 | 0.469 | 1.899 |
|  | CY | Cyprus | 0.301 | 0.686 | 0.782 | 1.523 |
|  | CZ | Czech Republic | 0.008 | 1.587 | 0.115 | 3.812 |
|  | EE | Estonia | 0.468 | 1.250 | 0.327 | 2.987 |
|  | HU | Hungary | 0.000 | 3.343 | 0.156 | 3.595 |
|  | LV | Latvia | 0.404 | 1.270 | 0.174 | 4.048 |
|  | LT | Lithuania | 0.778 | 1.072 | 0.388 | 2.525 |
|  | MT | Malta | 0.337 | 1.516 | 0.652 | 2.230 |
|  | PL | Poland | 0.258 | 1.196 | 0.235 | 2.651 |
|  | SK | Slovakia | 0.014 | 1.623 | 0.066 | 5.031 |
|  | SI | Slovenia | 0.757 | 1.082 | 0.444 | 2.256 |
|  | BG | Bulgaria | 0.673 | 1.082 | 0.215 | 3.004 |
|  | RO | Romania | 0.000 | 1.827 | 0.046 | 5.239 |
|  | HR | Croatia |  |  |  |  |
| Internet experience | How often do you use the internet for personal use in the following activities? Browsing websites | 1 = Never, 6 = Every day | 0.000 | 0.918 | 0.856 | 0.989 |
|  | ...Looking at content on social media network websites and apps (e.g., looking at text, images, videos on Facebook, Twitter, Instagram) | 1 = Never, 6 = Every day | 0.207 | 0.986 | 0.001 | 0.881 |
|  | ...Playing games online | 1 = Never, 6 = Every day | 0.157 | 1.011 | 0.345 | 1.029 |
| Perceived  prevalence  and risks | How widespread do you think the problem of online child sexual abuse is in your country? [3 categories] | Online abuse: Very or rather rare | 0.000 | 1.276 | 0.001 | 1.781 |
|  |  | Online abuse: Rather widespread | 0.000 | 1.176 | 0.493 | 0.895 |
|  |  | Online abuse: Very widespread |  |  |  |  |
|  | To what extent do you agree or disagree with the following statements? Children can safely use the internet without being exposed to harmful content [3 categories] | No harmful content: Strongly disagree | 0.001 | 0.847 | 0.046 | 1.380 |
|  |  | No harmful content: Rather disagree | 0.003 | 0.871 | 0.452 | 0.895 |
|  |  | No harmful content: Rather or strongly agree |  |  |  |  |
|  | ...Children can safely use the internet without being approached by adults seeking to harm them [3 categories] | No harmful adults: Strongly disagree | 0.000 | 0.820 | 0.000 | 0.231 |
|  |  | No harmful adults: Rather disagree | 0.019 | 1.111 | 0.000 | 0.513 |
|  |  | No harmful adults: Rather or strongly agree |  |  |  |  |
|  | Children are increasingly at risk online [3 categories] | Children increasingly at risk: Strongly or rather disagree | 0.000 | 1.953 | 0.000 | 6.594 |
|  |  | Children increasingly at risk: Rather agree | 0.000 | 1.320 | 0.001 | 1.558 |
|  |  | Children increasingly at risk: Strongly agree |  |  |  |  |

**Table 6.** Predictive power of multinomial regression models. Source: Authors’ analysis of Flash EB 532 data.

| **Pseudo R- Square** | **Model 1** | **Model 2** | **Model 3** | **Model 4** |
| --- | --- | --- | --- | --- |
| Cox and Snell | 0.031 | 0.059 | 0.063 | 0.094 |
| **Nagelkerke** | **0.040** | **0.076** | **0.082** | **0.122** |
| McFadden | 0.021 | 0.041 | 0.044 | 0.067 |

# Country-level data: Digital Economy and Society Index (DESI) and Augmented Human Development Index (AHDI)

**Table 7.** Country scores for DESI and AHDI. Source: [European Commission](https://digital-decade-desi.digital-strategy.ec.europa.eu/datasets/desi/charts) and [Our World in Data](https://ourworldindata.org/grapher/augmented-human-development-index).

| **Country** | **DESI** | **AHDI** |
| --- | --- | --- |
| Austria | 54.70 | 0.74 |
| Belgium | 50.30 | 0.74 |
| Bulgaria | 37.70 | 0.54 |
| Croatia | 47.50 | 0.63 |
| Cyprus | 48.40 | 0.71 |
| Czechia | 49.10 | 0.69 |
| Denmark | 69.30 | 0.80 |
| Estonia | 56.50 | 0.77 |
| Finland | 69.60 | 0.79 |
| France | 53.30 | 0.73 |
| Germany | 52.90 | 0.84 |
| Greece | 38.90 | 0.66 |
| Hungary | 43.80 | 0.53 |
| Ireland | 62.70 | 0.76 |
| Italy | 49.30 | 0.69 |
| Latvia | 49.70 | 0.69 |
| Lithuania | 52.70 | 0.70 |
| Luxembourg | 58.90 | - |
| Malta | 60.90 | 0.74 |
| Netherlands | 67.40 | 0.77 |
| Poland | 40.50 | 0.62 |
| Portugal | 50.80 | 0.64 |
| Romania | 30.60 | 0.55 |
| Slovakia | 43.40 | 0.69 |
| Slovenia | 53.40 | 0.71 |
| Spain | 60.80 | 0.69 |
| Sweden | 65.20 | 0.80 |
